# Cellulose-supported chiral rhodium nanoparticles as sustainable heterogeneous catalysts for asymmetric carbon–carbon bond-forming reactions†
†Electronic supplementary information (ESI) available: General procedures, materials, and instrumentation; synthesis, characterization and relevant spectra/charts; procedures and results for optimization and additional experiments. See DOI: 10.1039/c5sc02510a


**DOI:** 10.1039/c5sc02510a

**Published:** 2015-08-12

**Authors:** Tomohiro Yasukawa, Hiroyuki Miyamura, Shū Kobayashi

**Affiliations:** a Department of Chemistry , School of Science , The University of Tokyo , Hongo, Bunkyo-ku , Tokyo 113-0033 , Japan . Email: shu_kobayashi@chem.s.u-tokyo.ac.jp

## Abstract

Cellulose-supported chiral Rh nanoparticle (NP) catalysts have been developed. The Rh NPs, which were well dispersed on cellulose, catalyzed the asymmetric 1,4-addition of arylboronic acids to enones and enoates, one of the representative asymmetric carbon–carbon bond-forming reactions, in the presence of chiral diene ligands, providing the corresponding adducts in high yields with outstanding enantioselectivities without metal leaching. The solid-state NMR analysis of the chiral NP system directly suggested interactions between the Rh NPs and the chiral ligand on cellulose. This is the first example of using polysaccharide-supported chiral metal nanoparticles for asymmetric carbon–carbon bond-forming reactions.

## Abstract 2

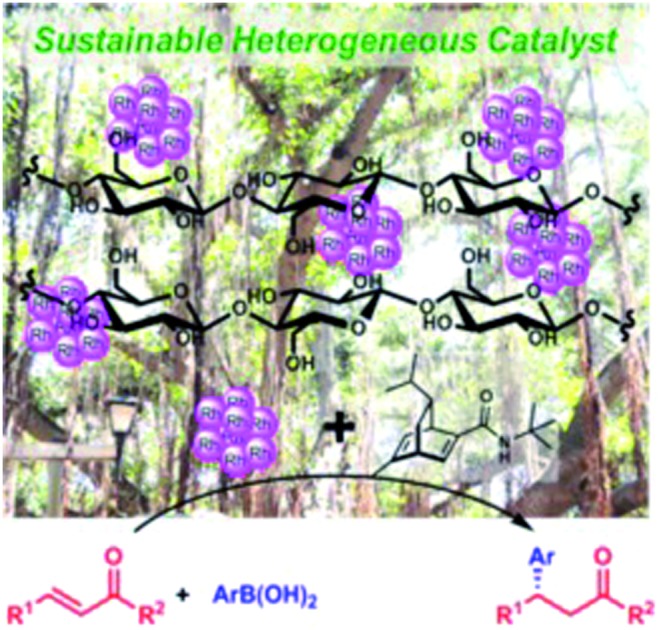
Cellulose-supported chiral Rh nanoparticle (NP) catalysts have been developed.

## Introduction

The development of heterogeneous chiral catalysts for asymmetric C–C bond-forming reactions is an imperative research subject for both academia^1^ and industry.^2^ The advantages of these systems, such as the ease of separation and reusability, can reduce the amount of scarce resources that are required, including precious metal-based catalysts. Although much effort has been devoted to this area in the last two decades, it is still challenging to obtain truly efficient and sustainable heterogeneous chiral catalysts. Recently, the great potential of chiral ligand-modified metal nanoparticles (NPs) as robust heterogeneous chiral catalysts has been demonstrated. Metal NPs can be deposited on solid supports to form stable heterogeneous catalysts, and such systems often show unique activities and selectivities that are distinct from those of homogeneous metal complexes.^3^ We have recently developed nanocomposites of a polystyrene-based copolymer with cross-linking moieties and a carbon black-incarcerated bimetallic Rh/Ag NP catalyst (PI/CB Rh/Ag), and found that PI/CB Rh/Ag catalyzed the asymmetric 1,4-addition of arylboronic acids to α,β-unsaturated carbonyl compounds in the presence of chiral diene ligands.^4^,^5^ Although high yields and enantioselectivities have been realized by using these catalyst systems and the robustness of PI/CB Rh/Ag has been demonstrated, Ag might not be crucial for the catalytic cycle and the polystyrene-based copolymer is originally derived from petroleum which is not a sustainable resource. Thus, the removal of the dopant as well as the use of more abundant and recyclable materials as supports are preferable for developing next-generation catalysts.

We focused on the use of cellulose as a support because this material has several advantages: (1) it has high stability and insolubility in common solvents, (2) it bears many hydroxyl groups that are expected to stabilize metal NPs effectively, and (3) it is abundant and is an environmentally benign biomass-derived material. Indeed, cellulose and its derivatives (polysaccharides) are very useful materials that are incorporated into a wide range of versatile products,^6^ and the application of such materials in catalytic systems has also been realized. An early example of the use of cellulose as a support for the immobilization of a Pd complex catalyst for a Tsuji–Trost reaction was reported in 2001.^7^ Kunitake and coworkers reported the immobilization of various mono-dispersed metal NPs on cellulose fibers *via* NaBH_4_-mediated reduction of cellulose fibers immersed in a metal salt.^8^ After these reports, the application of cellulose-supported metal NPs to catalysis was explored by many groups. For example, it was reported that Pd NPs catalyzed coupling reactions,^9^ hydrogenations,9*f* and Tsuji–Trost reactions,^10^ Au NPs catalyzed aerobic oxidations^11^ and reductions,^12^ and Cu NPs catalyzed C–N couplings,^13^ aza-Michael additions,^14^ and reductions.^15^ However, the applications of polysaccharide-supported catalysts to asymmetric transformations are limited to reports on the use of functionalized cellulose derivatives or chitosan as organocatalysts^16^ or as chiral ligands for metal complexes.^17^ To our knowledge, there have been no reports on catalytic asymmetric C-C bond formation using polysaccharide-supported metal NPs as heterogeneous catalysts.^18^ Herein, we report the first example of cellulose-supported chiral Rh NP catalysts as efficient and sustainable chiral catalyst systems for asymmetric 1,4-addition reactions.

## Results and discussion

Cellulose-supported Rh NP catalysts were prepared in THF using Rh_2_(OAc)_4_ and NaBH_4_ as the reductant, based on our previous methods that were used to immobilize metal NPs on supports such as polystyrene-based copolymers and polysilanes (Table 1).4b,^19^ The NPs were immediately deposited on cellulose as soon as the solution of Rh salt was added to the suspension of cellulose.^20^ Successive heating and washing with water and organic solvents afforded Rh-Cell **I**. By using this method, the Rh NPs were successfully immobilized on cellulose (entry 1). A bimetallic Rh/Ag NP catalyst was prepared from Rh_2_(OAc)_4_ and AgSbF_6_ using the same method, and a high loading of each metal was observed (entry 2). Scanning transmission electron microscopy (STEM) analysis of Rh-Cell **I** revealed that although several NPs were sometimes assembled, the size of each NP was relatively small (2–5 nm) and the NPs were well dispersed.^21^ In Rh/Ag-Cell, larger NPs were observed, indicating that the silver dopant caused aggregation in the case of cellulose-supported catalysts.^21^ Anhydrous RhCl_3_ was also examined as a Rh source for the preparation of the catalyst. Given the poor solubility of this Rh salt in common solvents, 1 M NaOH aq. was used (entry 3). STEM analysis of the obtained catalyst, Rh-Cell **II**, revealed better size distributions of the smaller NPs (*ca.* 3 nm).^21^ To clarify whether the use of RhCl_3_ or NaOH aq. mainly affected the size distribution, the catalyst was prepared from Rh_2_(OAc)_4_ in the presence of NaOH aq. (entry 4). Because Rh_2_(OAc)_4_ did not dissolve in 1 M NaOH aq., the latter was added first followed by the addition of a solution of Rh_2_(OAc)_4_ in THF to form Rh-Cell **III**. Based on the STEM images of the obtained catalyst,^21^ the size of NPs in Rh-Cell **III** were larger than that in Rh-Cell **II**. It was therefore evident that the choice of metal source was a major factor that determined the size distribution. When Rh/Cell **IV** was prepared from Rh_2_(OAc)_4_ in the presence of water instead of NaOH aq., a lower loading was obtained (entry 5) and no significant change in the size distribution of the nanoparticles was observed.^21^

**Table 1:** Preparation of the polysaccharide-supported Rh NP catalysts

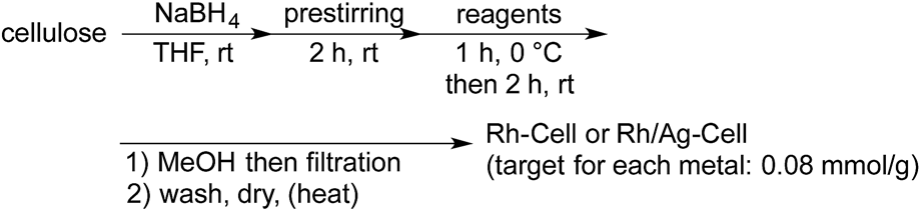			
Entry	Catalyst	Reagents	Rh/(Ag) loading^*a*^ (mmol g^–1^)
1	Rh-Cell **I**	Rh_2_(OAc)_4_ in THF	0.0622
2	Rh/Ag-Cell	Rh_2_(OAc)_4_ and AgSbF_6_ in THF	0.0693/0.0672
3	Rh-Cell **II**	RhCl_3_ in 1 M NaOH aq.	0.0651
4	Rh-Cell **III**	1 M NaOH aq., then Rh_2_(OAc)_4_ in THF	0.0651
5	Rh-Cell **IV**	H_2_O then Rh_2_(OAc)_4_ in THF	0.0554

^*a*^Determined *via* ICP analysis.

Notably, both the Rh NPs and Rh/Ag NPs were stabilized and immobilized on cellulose. Several control studies using partially methylated cellulose and d-glucose as the supports showed that similar metal loadings and STEM images to those of Rh-Cell were obtained in both supported catalysts.^21^ This observation suggests that there was no formation of strong bonds, such as covalent bonds or hydrogen bonds, between the Rh NPs and the supports, and that the Rh NPs might be stabilized and immobilized by interactions between the metals and oxygen atoms of cellulose and its derivatives.^8^

The catalytic activity of these NPs was then tested in the asymmetric 1,4-addition of phenylboronic acid (**2a**) to the aliphatic enone **1a** in the presence of the chiral diene **4a** (Table 2). Rh-Cell **I** showed a high catalytic performance, affording the desired product, **3aa**, in high yield and excellent enantiomeric excess (ee) and no metal leaching was observed (entry 1). The catalyst loading of Rh-Cell **I** could be reduced to 0.5 mol% without loss of yield, with a negligible level of metal leaching (entry 2). In contrast to the PI/CB system, the bimetallic Rh/Ag-Cell catalyst showed lower activity than Rh-Cell **I**, probably because of the larger size of NPs in Rh/Ag-Cell (entry 3). In spite of the formation of smaller NPs, a lower catalytic activity and a significant amount of metal leaching were observed in the reaction with Rh-Cell **II** (entry 4). Further heating or washing treatments of this catalyst failed to prevent the leaching.^21^ Rh-Cell **III** showed a good catalytic activity with 0.5 mol% catalyst loading and no metal leaching was observed (entry 5), whereas Rh-Cell **IV** suffered metal leaching under the same reaction conditions (entry 6), indicating that the addition of NaOH during the catalyst preparation is important not only to immobilize the Rh NPs at high loading but also to prevent metal leaching. Although the effect of NaOH is unclear, the negatively charged cellulose might be able to interact with Rh strongly and efficiently stabilize the NPs under the basic conditions; indeed, a slightly higher negative zeta potential under basic conditions than under neutral conditions was observed for cellulose by Haruta and coworkers.^11^ Given that the method of preparation of Rh-Cell **III** was relatively straightforward and considering that an excellent result was obtained in the model reaction, Rh-Cell **III** was established as the best catalyst. Further optimization^21^ using Rh-Cell **III** revealed that the use of the secondary amide-substituted chiral diene **4b**4*a* gave a higher ee and that the amount of chiral diene could be reduced to 0.05 mol% without affecting the yield or the extent of metal leaching (entry 7).

**Table 2:** Asymmetric 1,4-addition to the enone

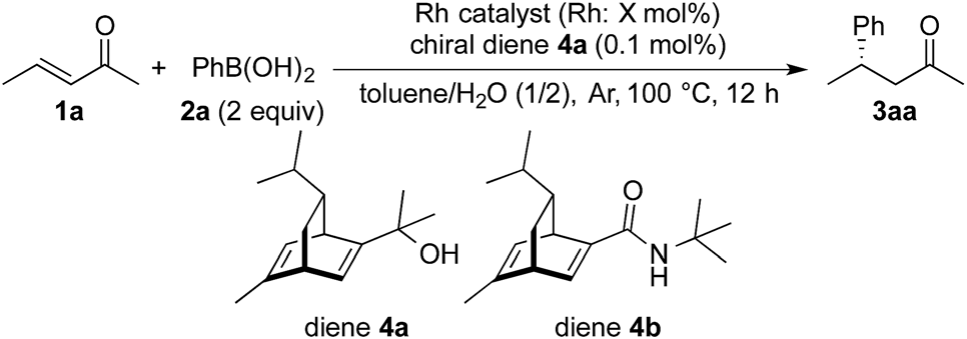					
Entry	Catalyst	*X* (mol%)	Yield^*a*^ (%)	ee^*b*^ (%)	Rh leaching^*c*^ (%)
1	Rh-Cell **I**	1.5	93	95	ND
2	Rh-Cell **I**	0.5	95	95	0.27
3	Rh/Ag-Cell	0.5	54	—	0.30
4	Rh-Cell **II**	0.5	65	—	3.0
5	Rh-Cell **III**	0.5	86	96	ND
6	Rh-Cell **IV**	0.5	84	—	0.78
7^*d*^	Rh-Cell **III**	0.5	91	98	ND

^*a*^Determined using GC analysis.

^*b*^Determined using HPLC analysis.

^*c*^Determined using ICP analysis (ND = not detected). The values express the percentage of the total amounts of Rh that was employed to the reaction. The detection limit of Rh leaching is 0.1% (entry 1), 0.22% (entry 5) and 0.17% (entry 7).

^*d*^The diene **4b** (0.05 mol%) was used instead of **4a**.

**Fig. 1:**
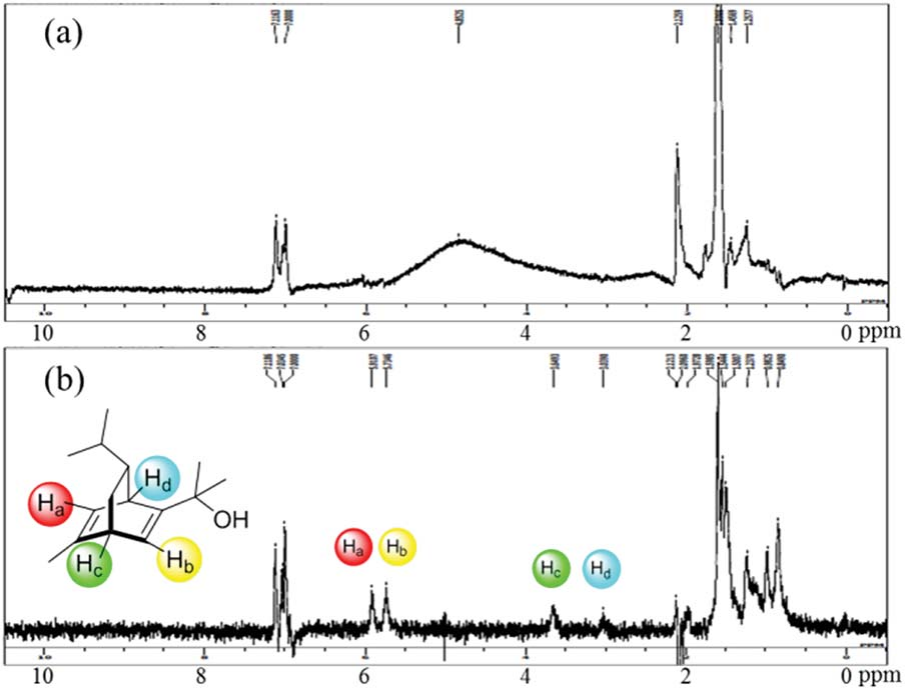
The SR-MAS analysis of the mixture of Rh-Cell and the chiral diene **4a** (a) with a diffusion filter and (b) with a diffusion filter and isotropic mixing.

A high yield and an excellent enantioselectivity were obtained by using the Rh-Cell catalyst and chiral diene **4a** or **4b**. For a high catalytic performance, an appropriate interaction of the Rh NPs, cellulose, and the chiral diene is crucial, and such interaction was suggested from swollen-resin magic angle spinning (SR-MAS) NMR^22^ analysis of the catalyst system. Solid-state NMR analysis with the addition of a solvent to swell the sample, so-called SR-MAS NMR, is a powerful tool to analyze heterogeneous systems. To confirm the formation of chiral NPs, characterization of the chiral diene adsorbed on the surface of the Rh NPs was undertaken in a mixture of Rh-Cell **I**, the chiral ligand **4a**, and toluene by conducting SR-MAS experiments, the pulse sequence of which consisted of a diffusion filter and isotropic mixing.^21^,^23^ The diffusion filter can suppress signals from molecules with high mobility such as a solvent and a chiral diene in the solution phase. The signal derived from **4a** (3–6 ppm) was completely suppressed by using the diffusion filter, and the signals derived from cellulose remained due to the lower diffusion coefficient of the polymer (Fig. 1a). Isotropic mixing can introduce an exchange between the remaining magnetization and nearby molecules with low mobilities, and the magnetization of cellulose is expected to exchange with nearby molecules that would be anchored in the catalyst. The signals derived from **4a** were enhanced with a diffusion filter followed by isotropic mixing (Fig. 1b). The same phenomenon was not observed for samples containing only cellulose and **4a** in toluene,^21^ which suggests that the molecules adsorbed on the Rh NPs were observed selectively using this method.

**Scheme 1:**
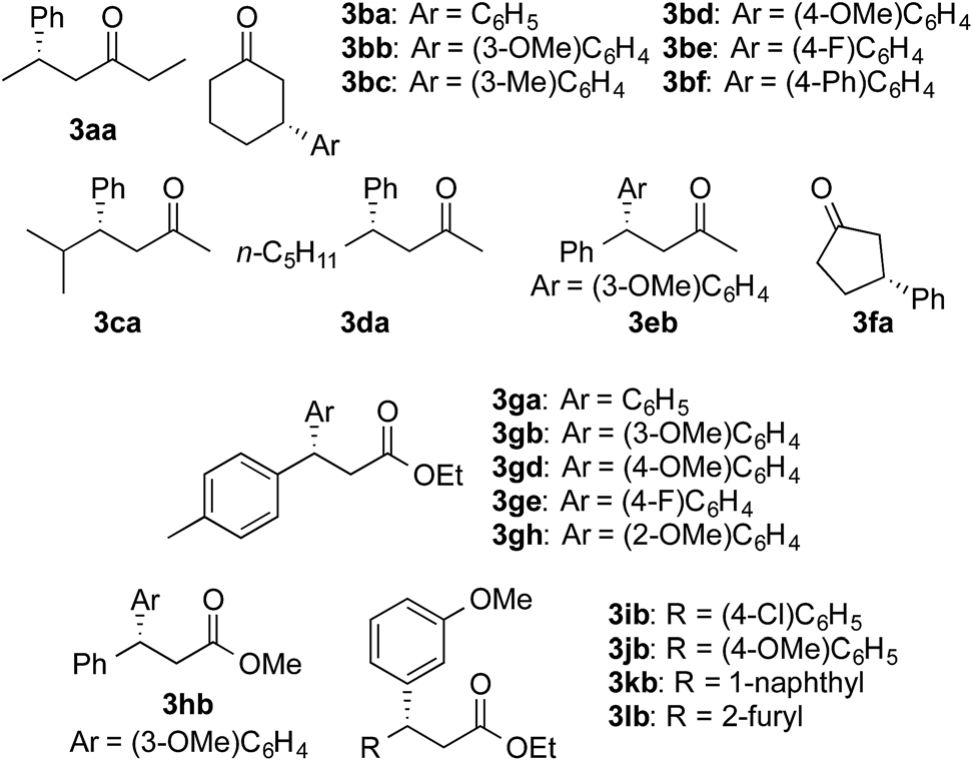
List of the products.

We then surveyed the substrate scope of the asymmetric 1,4-addition of arylboronic acids to enones with Rh-Cell **III** under the optimized conditions (Table 3, Scheme 1). The cyclic enone **1b** and the branched acyclic enone **1c** were smoothly converted into the products in high yields and excellent ee values with 1.5 equiv. **2a** (entries 2 and 3). Excellent yields and ee values were also observed in the reaction with the acyclic enone **1d**, aromatic enone **1e**, and cyclic enone with a five-membered ring **1f**, when the reaction time was prolonged to 20 h (entries 4–6). The substrate generality of the arylboronic acid part was examined by using **1b**. Irrespective of the substitution position, a wide range of arylboronic acids with either an electron-donating or electron-withdrawing group was suitable, and the reaction afforded the products in high yields and outstanding ee values (entries 7–11).

**Table 3:** Substrate scope of the asymmetric 1,4-addition to enones

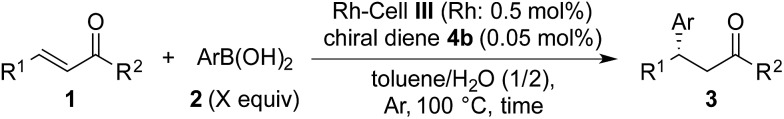					
Entry	Product	Time (h)	*X* (equiv.)	Yield^*a*^ (%)	ee^*b*^ (%)
1	**3aa**	12	2.0	95^*c*^	98
2	**3ba**	12	1.5	95	99
3	**3ca**	12	1.5	86	98
4	**3da**	20	2.0	94	98
5	**3eb**	20	2.0	87	96
6	**3fa**	20	1.5	90	99
7	**3bb**	20	1.5	83	99
8	**3bc**	20	1.5	84	97
9	**3bd**	20	1.5	89	98
10	**3be**	20	1.5	86	98
11	**3bf**	20	1.5	87	99

^*a*^Isolated yield.

^*b*^Determined using HPLC analysis.

^*c*^Determined using GC analysis.

The catalyst system with Rh-Cell **III** was also applied to the asymmetric 1,4-addition to α,β-unsaturated esters (Table 4, Scheme 1). When the use of the aromatic unsaturated ethyl ester **1g** was examined, the desired products were obtained in high yields and with excellent ee values under the same conditions after 20 h of reaction time with a wide range of arylboronic acids (entries 1–4). In the case of the *ortho*-substituted boronic acid **2h**, 1 equiv. of base was required to achieve a high yield (entry 5). The addition of base also promoted the reaction with the aromatic unsaturated methyl ester **1h** (entry 6). Aromatic unsaturated ethyl esters with substituents and substrates bearing a naphthyl group were smoothly converted into the products in high yields with excellent ee values (entries 7–9). Heteroarene-substituted substrates could be converted into the product in a high yield and an excellent ee in the presence of a base (entry 10).

**Table 4:** Substrate generality of the reaction with α,β-unsaturated esters

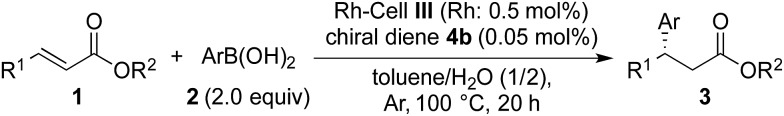				
Entry	Product	Additive	Yield^*a*^ (%)	ee^*b*^ (%)
1	**3ga**	—	96	98
2	**3gb**	—	93	99
3	**3gd**	—	92	98
4	**3ge**	—	81	99
5	**3gh**	1.0 equiv. K_2_CO_3_	81	>99
6	**3hb**	0.1 equiv. K_2_CO_3_	91	98
7^*c*^	**3ib**	—	81	99
8	**3jb**	—	91	98
9	**3kb**	—	95	99
10^*c*^	**3lb**	0.1 equiv. K_2_CO_3_	81	98

^*a*^Isolated yield.

^*b*^Determined using HPLC analysis.

^*c*^ **4b** (0.1 mol%) and **III** (Rh: 1.0 mol%) were used.

Rh-Cell **III** could be recovered by filtration and the reusability of the catalyst was tested in the reaction of **1a** with **2a**. The recovered catalyst, when washed with acidic media (THF/1 M TfOH aq., 99 : 1) between runs, maintained its catalytic activity to afford **3aa** in >95% yield over three cycles. A new portion of the chiral diene was employed every run and the enantioselectivity was 98% ee in all cases. It is possible that TfOH neutralizes and removes basic impurities.^24^ The Rh loading of the recovered catalyst after several runs was almost unchanged from that of the catalyst before use, indicating that no metal leaching occurred either during the reactions or upon washing in acidic media.

**Fig. 2:**
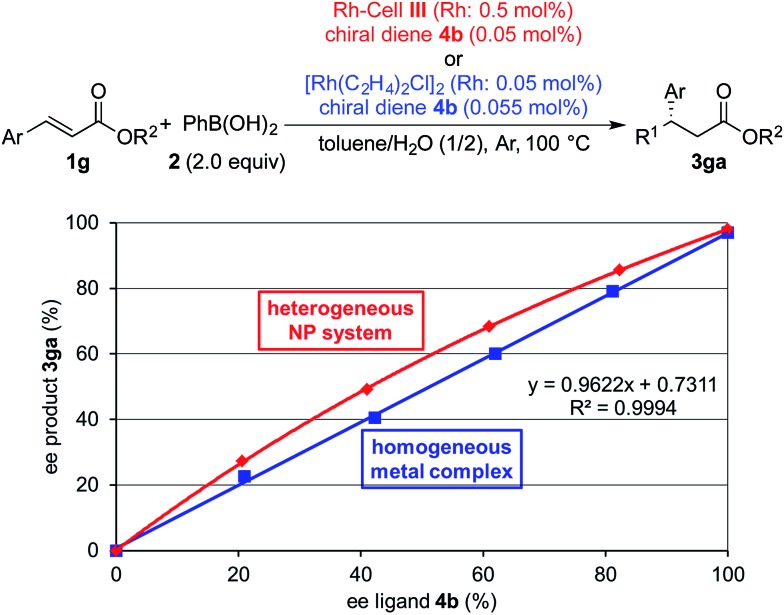
Non-linear effect analysis.

Finally, we performed nonlinear effect (NLE) analysis^25^ to obtain information about the nature of the active species in the current catalyst system. We previously discovered that a positive NLE was observed in the PI/CB Rh/Ag system, whereas a linear relationship between the enantiomeric excess of the ligand and that of the product was observed in the homogeneous metal complex system.4*a* These results clearly distinguished the nature of the active species involved in the two systems. In the case of the Rh-Cell system, a positive NLE similar to that in the PI/CB Rh/Ag system was observed, and the characteristic nature of the current cellulose-based heterogeneous NP system, that was distinct from the nature of the homogeneous metal complex system, was confirmed (Fig. 2).

## Conclusions

Cellulose, as a readily available and abundant material, was found to be an excellent support for Rh NP catalysts. The procedure used for the preparation of Rh-Cell is straightforward and Rh NPs were well dispersed over the cellulose. The combination of Rh-Cell with a chiral diene ligand showed an excellent catalytic performance for asymmetric 1,4-additions to enones and α,β-unsaturated esters, without the addition of silver, to afford the desired products in high yields with outstanding ee values without metal leaching. This is the first example of using polysaccharide-supported chiral metal nanoparticles for asymmetric carbon–carbon bond-forming reactions. We believe that this cellulose-supported chiral Rh NP catalyst system is a truly sustainable asymmetric catalyst system.

## Supplementary Material

Supplementary information
