# Supplementary material for: Cellulose-supported chiral rhodium nanoparticles as sustainable heterogeneous catalysts for asymmetric carbon–carbon bond-forming reactions
Source: Chem Sci. 2015 Aug 12;6(11):6224–9. doi: 10.1039/c5sc02510a (PMC6054049; doi:10.1039/c5sc02510a)
Supplement: Supplementary file 1 [file SC-006-C5SC02510A-s001.pdf]

Electronic Supplementary Material (ESI) for Chemical Science.
This journal is © The Royal Society of Chemistry 2015

Cellulose-Supported Rh Nanoparticles as Sustainable Heterogeneous Catalysts for Asymmetric 1,4-Addition of Arylboronic Acids to Enones and Enoates in the Presence of Chiral Dienes

Tomohiro Yasukawa, Hiroyuki Miyamura, and Shū Kobayashi*
Department of Chemistry, School of Science, The University of Tokyo, Hongo, Bunkyo-ku, Tokyo 113-0033, Japan.

Electronic Supplementary Information

Contents

1. General--S-1
2. Preparation of Rh/Cell ---S-3
3. Asymmetric 1,4-addition of arylboronic acids to enones or enoates-------------------S-5
4. Structure analysis--S-15
5. References---S-24

1. General

- JEOL JNM-ECA 500 or ECX 600 spectrometers were used for NMR measurement. Chloroform (δ = 7.24) was used as an internal standard for ^{1}H NMR and $CDCl_3$ (δ = 77.0) for ^{13}C NMR. Structures of known compounds were confirmed by comparison with commercially available compounds or data shown in literature.
- JEOL JNM-ECX 600 spectrometers and 3.2 mm FG-MAS probe were used for SR-MAS measurement. Toluene (δ = 7.00) was used as an internal standard.
- IR spectra were measured on a JASCO FT/IR-610 spectrometer.
- Preparative thin-layer chromatography was carried out using Wakogel B-5F.
- ICP analysis was performed on Shimadzu ICPS-7510 equipment.
- GC analysis. was performed on Shimadzu GC-2010 apparatus (**Condition A :** Column = GL Science, TCWAX, 0.25 mm ID, 0.25 μm, 60.0 m; Gas pressure: 214.2 kPa; Total flow: 90.6 mL/min; Column flow: 1.86 mL/min; Velocity: 30.8 cm/sec; Purge flow: 3.0 mL/min; Sprit ratio: 46.0; Injector: 250 ˚C, FID: 250 ˚C; Column program: starting from 50.0 ˚C, 10 min hold, 10 ˚C/min to 220 ˚C, 15 min hold) (**Condition B :** column = J & W SCIENTIFIC DB-1 0.25 mm ID, 0.25 μm, 60.0 m; Gas pressure: 157.5 kPa, Total flow: 41.3 mL/min, Column folw: 0.93 mL/min, Velocity: 21.1 cm/sec; Purge flow: 3.0 mL/min; Sprit ratio: 40.1; Injector: 300 °C, FID: 300 °C; Column program: starting from 100 ˚C, 20 ˚C/min to 300 ˚C,

10 min hold).

- HPLC analysis was performed on Shimadzu LC-20AB, SPD-M20A (or SPD-20A) and DGU-20A$_3$.
- The absolute configuration of reported compounds was determined by comparison to literature and that of other products was assumed by analogy.
- STEM/EDS images were obtained using a JEOL JEM-2100F instrument operated at 200 kV. All STEM specimens were prepared by placing a drop of the solution on carbon-coated Cu grids and allowed to dry in air (without staining).
- $Rh_2(OAc)_4$ was purchased from Strem Chemical Inc.
- $AgSbF_6$ complex was purchased from Sigma-Aldrich Co., Ltd..
- $NaBH_4$ was purchased from Wako Pure Chemical Company and recrystallized from diglyme by heating according to the literature[1] and stored in a glove box.
- Ketjen black EC300J was purchased from Lion Corporation.
- Cellulose, Powder, through 38μm (400mesh) was purchased from Wako Pure Chemical Company and dried *in vacuo* using heat gun before use.
- Toluene was purchased in dried grade from Wako Pure Chemical Company and used without further purification.
- Deionized water from a MILLIPORE MilliQ machine (Gradient A 10) was used as solvent without further treatment.
- Enones were purchased from Tokyo Kasei Kogyo Co., Ltd..
- Aliphatic unsaturated esters and cinnamate were purchased from Tokyo Kasei Kogyo Co., Ltd..
- Aryl unsaturated esters except cinnamate were prepared by following the literature.[2]
- Arylboronic acids were purchased from Wako Pure Chemical Company or prepared from the corresponding Grignard reagent. The ratio of boronic acid to boroxine was determined by ^{1}H NMR analysis before use.
- Chiral diene **4a** and **4b** were prepared by following the literatures.[3]
- PI/CB Rh was prepared by following the literature.[4]
- Asymmetric 1,4-addition reactions were conducted with Carousel™.
- Unless otherwise stated, all reactions were carried out under argon atmosphere.
- 0.45 μm PTFE membrane filter (Whatman™ cat. No. 6784-2504) was used for filtration of solid catalysts.

2. Preparation of Rh-Cell

2-1. Preparation of Rh-Cell I (Table 1, entry 1): To a suspension of cellulose (1.2 g) in THF (4 mL), a solution of $NaBH_4$ (72.6 mg, 1.92 mmol) in diglyme (1.5 mL) was added at room temperature, and the mixture was stirred for 2 h at the same temperature under Ar atmosphere. To this suspension was slowly added a solution of rhodium(II) acetate dimer (21.2 mg, 0.048 mmol) in THF (1.5 mL). The mixture was stirred for 1 h at 0 ˚C and the stirring was continued for 2 h at room temperature. Methanol (50 mL) was slowly added to the mixture at room temperature. The catalyst was filtered, washed with methanol and water and dried *in vacuo*. The catalyst was heated at 100 ˚C for 5 h without solvent under air and stirred in 1:1 ratio of THF/water co-solvent for a couple of hours at room temperature. The catalyst was filtered, washed with water, THF and acetone and dried *in vacuo* to afford Rh-Cell **I**. Rh-Cell **I** (10-20 mg) was heated in a mixture of sulfuric acid and nitric acid at 200 ˚C, and the mixture was cooled to room temperature. The amount of Rh in the resulting solution was measured by ICP analysis to determine the loading of Rh.

2-2. Preparation of Rh/Ag-Cell (Table 1, entry 2): To a suspension of cellulose (1.2 g) in THF (4 mL), a solution of $NaBH_4$ (72.6 mg, 1.92 mmol) in diglyme (1.5 mL) was added at room temperature, and the mixture was stirred for 2 h at room temperature under Ar atmosphere. To this suspension was slowly added a mixture of rhodium(II) acetate dimer (21.2 mg, 0.048 mmol) and $AgSbF_6$ (33.0 mg, 0.096 mmol) in THF (1.5 mL). The mixture was stirred for 1 h at 0 ˚C and the stirring was continued for 2 h at room temperature. Methanol (50 mL) was slowly added to the mixture at room temperature. The catalyst was filtered, washed with methanol and water and dried *in vacuo*. The catalyst was heated at 100 ˚C for 5 h without solvent under air and stirred in 1:1 ratio of THF/water co-solvent for a couple of hours at room temperature. The catalyst was filtered, washed with water, THF and acetone and dried *in vacuo* to afford Rh/Ag-Cell. Rh/Ag-Cell (10-20 mg) was heated in a mixture of sulfuric acid and nitric acid at 200 ˚C, and the mixture was cooled to room temperature. The amounts of Rh and Ag in the resulting solution were measured by ICP analysis to determine the loading of Rh and Ag.

2-3. Preparation of Rh-Cell II (Table 1, entry 3): To a suspension of cellulose (1.2 g) in THF (4 mL), a solution of $NaBH_4$ (72.6 mg, 1.92 mmol) in diglyme (1.5 mL) was added at room temperature, and the mixture was stirred for 2 h at the same temperature under Ar atmosphere. To this suspension was slowly added a solution of rhodium(III)

chloride (20.1 mg, 0.096 mmol) in 1 M NaOH aq. (2.5 mL). The mixture was stirred for 1 h at 0 ˚C and the stirring was continued for 2 h at room temperature. Methanol (50 mL) was slowly added to the mixture at room temperature. The catalyst was filtered, washed with methanol, water and acetone and stirred in 1:1 ratio of THF/water co-solvent for a couple of hours at room temperature. The catalyst was filtered, washed with water, THF and acetone and dried *in vacuo* to afford Rh-Cell **II**. Rh-Cell **II** (10-20 mg) was heated in a mixture of sulfuric acid and nitric acid at 200 ˚C, and the mixture was cooled to room temperature. The amount of Rh in the resulting solution was measured by ICP analysis to determine the loading of Rh.

2-4. Preparation of Rh-Cell III (Table 1, entry 4): To a suspension of cellulose (1.2 g) in THF (4 mL), a solution of $NaBH_4$ (72.6 mg, 1.92 mmol) in diglyme (1.5 mL) were added at room temperature, and the mixture was stirred for 2 h at the same temperature under Ar atmosphere. To this suspension, 1 M NaOH aq. (2.5 mL) was added, followed by a slow addition of a solution of rhodium(II) acetate dimer (21.2 mg, 0.048 mmol) in THF (1.5 mL). The mixture was stirred for 1 h at 0 ˚C and the stirring was continued for 2 h at room temperature. Methanol (50 mL) was slowly added to the mixture at room temperature. The catalyst was filtered, washed with methanol, water and acetone and stirred in 1:1 ratio of THF/water co-solvent for a couple of hours at room temperature. The catalyst was filtered, washed with water, THF and acetone and dried *in vacuo* to afford Rh-Cell **III**. Rh-Cell **III** (10-20 mg) was heated in a mixture of sulfuric acid and nitric acid at 200 ˚C, and the mixture was cooled to room temperature. The amount of Rh in the resulting solution was measured by ICP analysis to determine the loading of Rh and B. No boron was detected by ICP analysis.

2-5. Preparation of Rh-Cell IV (Table 1, entry 5): To a suspension of cellulose (1.2 g) in THF (4 mL), a solution of $NaBH_4$ (72.6 mg, 1.92 mmol) in diglyme (1.5 mL) was added at room temperature, and the mixture was stirred for 2 h at the same temperature under Ar atmosphere. To this suspension, water (2.5 mL) was added, followed by a slow addition of a solution of rhodium(II) acetate dimer (21.2 mg, 0.048 mmol) in THF (1.5 mL). The mixture was stirred for 1 h at 0 ˚C and the stirring was continued for 2 h at room temperature. Methanol (50 mL) was slowly added to the mixture at room temperature. The catalyst was filtered, washed with methanol, water and acetone and stirred in 1:1 ratio of THF/water co-solvent for a couple of hours at room temperature. The catalyst was filtered, washed with water, THF and acetone and dried *in vacuo* to afford Rh-Cell **IV**. Rh-Cell **IV** (10-20 mg) was heated in a mixture of sulfuric acid and

nitric acid at 200 ˚C, and the mixture was cooled to room temperature. The amount of Rh in the resulting solution was measured by ICP analysis to determine the loading of Rh.

3. Asymmetric 1,4-addition of arylboronic acids to enones or enoates

3-1. A typical procedure of asymmetric 1,4-addition of arylboronic acids to enones or enoates catalyzed by Rh-Cell (Table 4, entry 2): Cyclohex-2-en-1-one **1b** (28.8 mg, 0.3 mmol), phenylboronic acid (a mixture of boroxine and boronic acid, 64.5 mg, 0.6 mmol as B), Rh-Cell **III** (Rh: 0.5 mol%), which was heated at 150 ˚C for 2 h under air without solvent just before use (this heating treatment gave a good reproducibility of high catalytic performance), and chiral diene **4b** (0.01 mL of 3.9 mg/mL solution of toluene), toluene (0.365 mL) and water (0.75 mL) were combined in a Carousel^{TM} tube. The mixture was stirred for 12 h under Ar atmosphere at 100 ˚C. If necessary, ethylbenzene (15~20 mg) as an internal standard was added to the mixture, and an aliquot of the reaction mixture (~0.02 mL) was filtered through a silica gel packed disposable Pasteur pipette and washed with ethyl acetate to inject the GC analysis. Diethyl ether was added to the mixture and the solid catalyst was removed by filtration. The mixture was transferred to a separating funnel and the organic layers were washed with water and dried over sodium sulfate. After that, the solvents were removed *in vacuo* and the residue was purified by preparative TLC to afford ethyl (*R*)-3-phenylcyclohexan-1-one **3ba** (49.6 mg, 95% yield). The ee value of the product was determined by chiral HPLC analysis.

(*S*)-5-phenylhexan-3-one (3aa)[4]:

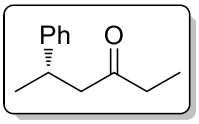

^{1}H NMR (CDCl$_3$, 600 MHz) δ = 0.97 (t, 3H, J = 7.2 Hz), 1.24 (d, 3H, J = 6.9 Hz), 2.25-2.35 (m, 2H), 2.61 (dd, 1H, J = 15.8, 7.6 Hz), 2.70 (dd, 1H, J = 16.1, 6.5 Hz), 3.28-3.32 (m, 1H), 7.15-7.19 (m, 3H), 7.27 (t, 2H, J = 7.6 Hz); ^{13}C NMR (CDCl$_3$, 150 MHz) δ = 7.6, 21.9, 35.5, 36.6, 50.8, 126.2, 126.7, 128.5, 146.3, 210.4. The ee value of the product was determined on Daicel Chiralpak AD-3 column with hexane/2-propanol = 99:1, flow = 0.5 mL/min by HPLC analysis. Retention times: 10.9 min [(*S*)-enantiomer], 12.1 min [(*R*)-enantiomer], 98% ee.

(*R*)-3-phenylcyclohexanone (3ba)[4]:

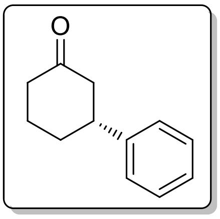

^{1}H NMR ($CDCl_3$, 500 MHz) δ = 1.73-1.88 (m, 2H), 2.06-2.16 (m, 2H), 2.33-2.60 (m, 4H), 2.96-3.03 (m, 1H), 7.20-7.23 (m, 3H), 7.30-7.33 (m, 2H); ^{13}C NMR ($CDCl_3$, 150 MHz) δ = 25.5, 32.7, 41.1, 44.7, 48.9, 126.5, 126.6, 128.6, 144.3, 210.9. The ee value of the product was determined on Daicel Chiralpak AD column with hexane/2-propanol = 49/1, flow = 0.5 mL/min by HPLC analysis. Retention times: 18.9min [(*S*)-enantiomer], 22.7 min [(*R*)-enantiomer], 99% ee.

(*R*)-3-(3-methoxyphenyl)cyclohexanone (3bb)[4]:

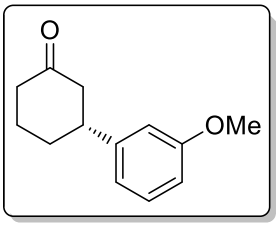

^{1}H NMR ($CDCl_3$, 500 MHz) δ = 1.71-1.87 (m, 2H), 2.06-2.16 (m, 2H), 2.32-2.60 (m, 4H), 2.97 (tt, 1H, *J* = 11.9, 3.7 Hz), 3.79 (s, 3H), 6.75-6.80 (m, 3H), 7.21-7.24 (m, 1H); ^{13}C NMR ($CDCl_3$, 125 MHz) δ = 25.5, 32.7, 41.2, 44.8, 48.9, 55.2, 111.7, 112.7, 118.9, 129.7, 146.0, 159.9, 210.8. The ee value of the product was determined on Daicel Chiralpak AD-H column with hexane/2-propanol = 49:1, flow = 0.5 mL/min by HPLC analysis. Retention times: 33.6 min [(*S*)-enantiomer], 35.4 min [(*R*)-enantiomer], 99% ee.

(*R*)-3-(*m*-tolyl)cyclohexanone (3bc)[4]:

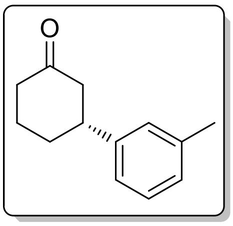

^{1}H NMR ($CDCl_3$, 600 MHz) δ = 1.73-1.81 (m, 2H), 2.04-2.16 (m, 2H), 2.33 (s, 3H), 2.31-2.59 (m, 4H), 2.95 (tt, 1H, *J* = 11.9, 3.9 Hz), 6.99-7.04 (m, 3H), 7.20 (t, 1H, *J* = 7.6 Hz); ^{13}C NMR ($CDCl_3$, 125 MHz) δ =21.4, 25.5, 32.8, 41.2, 44.7, 48.9, 123.5, 127.3, 127.4, 128.5, 138.2, 144.3, 210.9. The ee value of the product was determined on Daicel Chiralpak AS-H column with hexane/2-propanol = 49:1, flow = 1.0 mL/min by HPLC analysis. Retention times: 18.9 min [(*S*)-enantiomer], 22.7 min [(*R*)-enantiomer], 97% ee.

(*R*)-3-(4-methoxyphenyl)cyclohexanone (3bd)[4]:

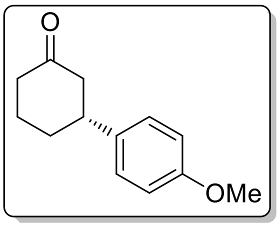

^{1}H NMR ($CDCl_3$, 600 MHz) δ = 1.70-1.83 (m, 2H), 2.03-2.13 (m, 2H), 2.32-2.57 (m, 4H), 2.95 (tt, 1H, *J* = 11.7, 3.9 Hz), 3.78 (s, 3H), 6.83-6.86 (m, 2H), 7.11-7.13 (m, 2H); ^{13}C NMR ($CDCl_3$, 150 MHz) δ = 25.5, 33.0, 41.2, 44.0, 49.2, 55.3, 114.1, 127.5, 136.6, 158.3, 211.0. The ee value of the product was determined on Daicel Chiralpak AD-H column with hexane/2-propanol = 49:1, flow =

0.5 mL/min by HPLC analysis. Retention times: 28.2 min [(*S*)-enantiomer], 29.7 min [(*R*)-enantiomer], 97% ee.

(*R*)-3-(4-fluorophenyl)cyclohexanone (3be)[4]:

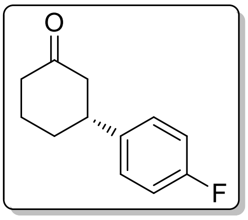

^{1}H NMR ($CDCl_3$, 500 MHz) δ = 1.71-1.84 (m, 2H), 2.04-2.15 (m, 2H), 2.32-2.58 (m, 4H), 2.95-3.01 (m, 1H), 6.97-7.01 (m, 2H), 7.13-7.17 (m, 2H); ^{13}C NMR ($CDCl_3$, 125 MHz) δ = 25.4, 32.9, 41.1, 44.0, 49.1, 115.4 (d, 1C, *J* = 21.5 Hz), 128.0 (d, 1C, *J* = 7.2 Hz), 140.1, 161.6 (d, 1C, *J* = 244.4 Hz), 210.5. The ee value of the product was determined on Daicel Chiralpak AS-H column with hexane/2-propanol = 49:1, flow = 1.0 mL/min by HPLC analysis. Retention times: 29.9 min [(*S*)-enantiomer], 33.9 min [(*R*)-enantiomer], 98% ee.

(*R*)-3-([1,1'-biphenyl]-4-yl)cyclohexanone (3bf)[4]:

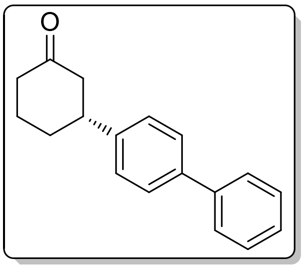

^{1}H NMR ($CDCl_3$, 500 MHz) δ = 1.75-1.92 (m, 2H), 2.10-2.18 (m, 2H), 2.35-2.64 (m, 4H), 3.02-3.07 (m, 1H), 7.28 (d, 2H, *J* = 7.9 Hz), 7.32 (t, 1H, *J* = 7.4 Hz), 7.41 (t, 2H, *J* = 7.9 Hz), 7.53-7.57 (m, 4H); ^{13}C NMR ($CDCl_3$, 150 MHz) δ = 25.5, 32.8, 41.2, 44.4, 48.9, 126.96, 126.98, 127.2, 127.4, 128.7, 139.7, 140.8, 143.4, 210.8. The ee value of the product was determined on Daicel Chiralpak AS-H column with hexane/2-propanol = 49:1, flow = 1.0 mL/min by HPLC analysis. Retention times: 31.3 min [(*R*)-enantiomer], 44.5 min [(*S*)-enantiomer], 98% ee.

(*R*)-5-methyl-4-phenylhexan-2-one (3ca)[4]:

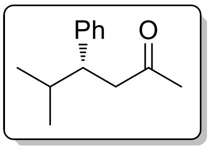

^{1}H NMR ($CDCl_3$, 500 MHz) δ = 0.73 (d, 3H, *J* = 6.8 Hz), 0.91 (d, 3H, *J* = 6.8 Hz), 1.78-1.83 (m, 1H), 1.95 (s, 3H), 2.73-2.81 (m, 2H), 2.90 (td, 1H, *J* = 8.2, 5.7 Hz), 7.11-7.17 (m, 2H), 7.23-7.26 (m, 3H); ^{13}C NMR ($CDCl_3$, 150 MHz) δ = 20.3, 20.7, 30.5, 33.3, 47.6, 48.1, 126.2, 128.1, 128.2, 143.3, 208.2. The ee value of the product was determined on Daicel Chiralpak OD-H column with hexane/2-propanol = 49:1, flow = 0.5 mL/min by HPLC analysis. Retention times: 12.7 min [(*R*)-enantiomer], 14.3 min [(*S*)-enantiomer], 98% ee.

(*S*)-4-phenylnonan-2-one (3da)[4]:

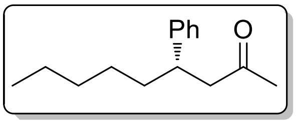

^{1}H NMR ($CDCl_3$, 500 MHz) δ = 0.80 (t, 3H, *J* = 7.1 Hz), 1.08-1.22 (m, 6H), 1.50-1.62 (m, 2H), 1.99 (s, 3H), 2.64-2.73 (m, 2H), 3.06-3.12 (m, 1H), 7.14-7.18 (m, 3H), 7.26 (t, 2H, *J* = 7.7 Hz); ^{13}C NMR ($CDCl_3$, 100 MHz) δ = 14.0, 22.5, 27.0, 30.6, 31.7, 36.4, 41.3, 50.9, 126.3, 127.4, 128.4, 144.6, 208.0. The ee value of the product was determined on Daicel Chiralpak OJ column with hexane/2-propanol = 99:1, flow = 0.25 mL/min by HPLC analysis. Retention times: 26.5 min [(*S*)-enantiomer], 29.1 min [(*R*)-enantiomer], 98% ee.

(*R*)-4-(3-methoxyphenyl)-4-phenylbutan-2-one (3eb)[3c]:

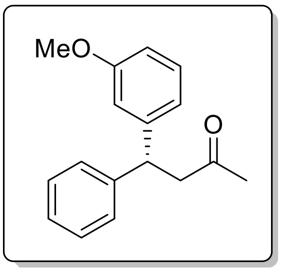

^{1}H NMR ($CDCl_3$, 600 MHz) δ = 2.06 (s, 3H), 3.15 (d, 2H, *J* = 7.6 Hz), 3.74 (s, 3H), 4.54 (t, 1H, *J* = 7.6 Hz), 6.70 (dd, 1H, *J* = 7.9. 2.4 Hz), 6.75 (s, 1H), 6.80 (d, 1H, *J* = 7.6 Hz), 7.14-7.26 (m, 6H); ^{13}C NMR ($CDCl_3$, 150 MHz) δ = 30.6, 46.0, 49.6, 55.1, 111.4, 113.9, 120.0, 126.4, 127.6, 128.6, 129.5, 143.7, 145.5, 159.7, 206.7. The ee value of the product was determined on Daicel Chiralcel OD-H column with hexane/2-propanol = 9:1, flow = 1.0 mL/min by HPLC analysis. Retention times: 14.8 min [(*R*)-enantiomer], 18.2 min [(*S*)-enantiomer], 96% ee.

(*R*)-3-phenylcyclopentanone (3fa)[4]:

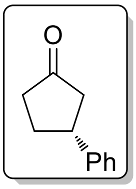

^{1}H NMR ($CDCl_3$, 500 MHz) δ = 1.96-2.05 (m, 1H), 2.27-2.38 (m, 2H), 2.42-2.50 (m, 2H), 2.65-2.70 (m, 1H), 3.40-3.47 (m, 1H), 7.24-7.27 (m, 3H), 7.34-7.37 (m, 2H); ^{13}C NMR ($CDCl_3$, 125 MHz) δ = 31.1, 38.8, 42.2, 45.7, 126.7, 128.6, 143.1, 218.2. The ee value of the product was determined on Daicel Chiralpak AS-H column with hexane/2-propanol = 99:1, flow = 0.5 mL/min by HPLC analysis. Retention times: 59.4 min [(*R*)-enantiomer], 64.8 min [(*S*)-enantiomer], 99% ee.

Ethyl (*S*)-3-phenyl-3-(*p*-tolyl)propanoate (3ga)[3c]:

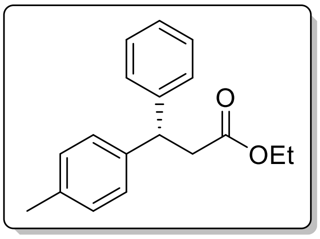

^{1}H NMR ($CDCl_3$, 500 MHz) δ = 1.09 (t, 3H, *J* = 7.1 Hz), 2.27 (s, 3H), 3.01 (d, 2H, *J* = 8.5 Hz), 4.01 (q, 2H, *J* = 7.0 Hz), 4.49 (t, 1H, *J* = 7.9 Hz), 7.05-7.17 (m, 5H), 7.20-7.26 (m, 4H); ^{13}C NMR ($CDCl_3$, 125 MHz) δ = 14.0, 20.9, 40.9, 46.7, 60.4, 126.4, 127.5, 127.6, 128.5, 129.2, 136.0, 140.5,

143.7, 171.8. The ee value of the product was determined on Daicel Chiralcel OD-H column with hexane/2-propanol = 99:1, flow = 1.0 mL/min by HPLC analysis. Retention times: 7.8 min [(*R*)-enantiomer], 12.5 min [(*S*)-enantiomer], 98% ee.

Ethyl (*R*)-3-(3-methoxyphenyl)-3-(*p*-tolyl)propanoate (3gb)[3c]:

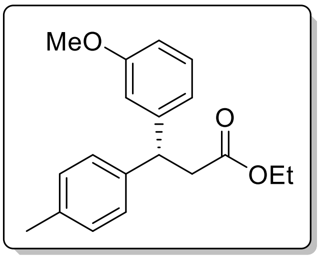

^{1}H NMR ($CDCl_3$, 500 MHz) δ = 1.10 (t, 3H, *J* = 7.4 Hz), 2.27 (s, 3H), 2.99 (d, 2H, *J* = 7.9 Hz), 3.74 (s, 3H), 4.02 (q, 2H, *J* = 7.2 Hz), 4.46 (t, 1H, 7.9 Hz), 6.70 (dd, 1H, *J* = 8.2, 2.6 Hz), 6.76 (d, 1H, *J* = 2.0 Hz), 6.81 (d, 1H, *J* = 7.4 Hz), 7.06 (d, 2H, *J* = 7.9 Hz), 7.11 (d, 2H, *J* = 7.9 Hz), 7.16 (t, 1H, *J* = 7.9 Hz); ^{13}C NMR ($CDCl_3$, 100 MHz) δ = 14.0, 20.9, 40.8, 46.7, 55.1, 60.4, 111.5, 113.7, 112.0, 127.5, 129.2, 129.4, 136.0, 140.3, 145.3, 159.6, 171.8. The ee value of the product was determined on Daicel Chiralcel OD-H column with hexane/2-propanol = 99:1, flow = 0.5 mL/min by HPLC analysis. Retention times: 27.0 min [(*S*)-enantiomer], 38.4 min [(*R*)-enantiomer], 99% ee.

Ethyl (*R*)-3-(4-methoxyphenyl)-3-(*p*-tolyl)propanoate (3gd)[3c]:

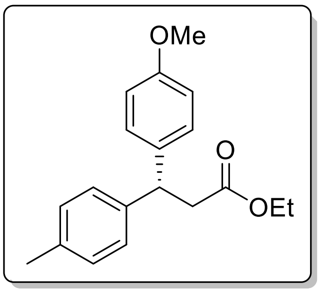

^{1}H NMR ($CDCl_3$, 600 MHz) δ = 1.11 (t, 3H, *J* = 7.6 Hz), 2.28 (s, 3H), 2.98 (d, 2H, *J* = 7.6 Hz), 3.74 (s, 3H), 4.02 (q, 2H, *J* = 7.1 Hz), 4.45 (t, 1H, *J* = 7.9 Hz), 6.79-6.81 (m, 2H), 7.06-7.14 (m, 6H); ^{13}C NMR ($CDCl_3$, 150 MHz) δ = 14.1, 20.9, 41.1, 45.9, 55.2, 60.3, 113.9, 127.4, 128.6, 129.2, 135.9, 140.9, 158.1, 171.9. The ee value of the product was determined on Daicel Chiralcel OD-H column with hexane/2-propanol = 99:1, flow = 0.5 mL/min by HPLC analysis. Retention times: 22.5 min [(*S*)-enantiomer], 25.7 min [(*R*)-enantiomer], 98% ee.

Ethyl (*R*)-3-(4-fluorophenyl)-3-(*p*-tolyl)propanoate (3ge)[3c]:

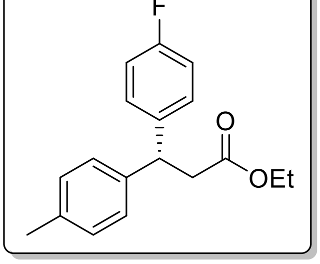

^{1}H NMR ($CDCl_3$, 500 MHz) δ = 1.10 (t, 3H, *J* = 7.4 Hz), 2.28 (s, 3H), 2.98 (d, 2H, *J* = 8.5 Hz), 4.02 (q, 2H, *J* = 7.0 Hz), 4.48 (t, 1H, *J* = 7.9 Hz), 6.92-6.96 (m, 2H), 7.08 (s, 4H), 7.16-7.19 (m, 2H); ^{13}C NMR ($CDCl_3$, 125 MHz) δ = 14.1, 20.9, 41.0, 46.0, 60.4, 115.3 (d, 1C, *J* = 20.3 Hz), 127.4, 129.1 (d, 1C, *J* = 8.4 Hz),129.3 136.2, 139.5 (d, 1C, *J* = 2.4 Hz), 140.4, 161.5, (d, 1C, *J* = 244.4 Hz), 171.7. The ee value of the product was determined on Daicel Chiralpak AS-H column with

hexane/2-propanol = 99:1, flow = 0.25 mL/min by HPLC analysis. Retention times: 31.5 min [(*S*)-enantiomer], 32.7 min [(*R*)-enantiomer], 99% ee.

Ethyl (*R*)-3-(2-methoxyphenyl)-3-(*p*-tolyl)propanoate (3gh):

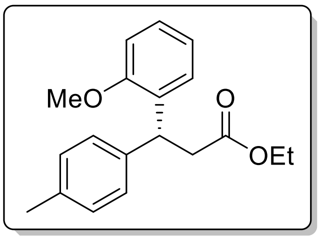

^{1}H NMR ($CDCl_3$, 500 MHz) δ = 1.09 (t, 3H, *J* = 7.4 Hz), 2.27 (s, 3H), 3.00 (dq, 2H, *J* = 27.8, 7.7 Hz), 3.77 (s, 3H), 4.01 (q, 2H, *J* = 7.0 Hz), 4.89 (t, 1H, *J* = 7.9 Hz), 6.81 (d, 1H, *J* = 7.9 Hz), 6.87 (t, 1H, *J* = 7.7 Hz), 7.04 (d, 2H, *J* = 7.9 Hz), 7.12-7.16 (m, 4H); ^{13}C NMR ($CDCl_3$, 125 MHz) δ = 14.1, 21.0, 39.8, 40.0, 55.4, 60.2, 110.9, 120.5, 127.5, 127.78, 127.80, 128.9, 132.2, 135.6, 140.3, 156.9, 172.1. The ee value of the product was determined on Daicel Chiralpak AS-H column with hexane/2-propanol = 99:1, flow = 1.0 mL/min by HPLC analysis. Retention times: 8.2 min [(*S*)-enantiomer], 9.3 min [(*R*)-enantiomer], >99.5% ee.

Methyl (*R*)-3-(3-methoxyphenyl)-3-phenylpropanoate (3hb)[3c]:

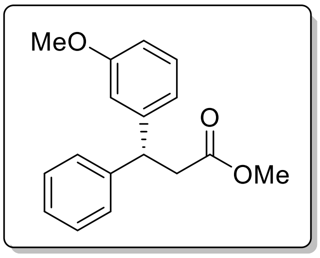

^{1}H NMR ($CDCl_3$, 600 MHz) δ = 3.03 (d, 2H, *J* = 8.3 Hz), 3.57 (s, 3H), 3.74 (s, 3H), 4.51 (t, 1H, *J* = 7.9 Hz), 6.70-6.72 (m, 1H), 6.75 (s, 1H), 6.81 (d, 1H, *J* = 7.6 Hz), 7.15-7.27 (m, 6H); ^{13}C NMR ($CDCl_3$, 150 MHz) δ = 40.5, 47.0, 51.6, 55.1, 111.6, 113.8, 120.0, 126.6, 127.6, 128.6, 129.5, 143.3, 145.1, 159.7, 172.2. The ee value of the product was determined on Daicel Chiralpak AS-H column with hexane/2-propanol = 99:1, flow = 1.0 mL/min by HPLC analysis. Retention times: 16.4 min [(*R*)-enantiomer], 19.7 min [(*S*)-enantiomer], 98% ee.

Ethyl (*R*)-3-(4-chlorophenyl)-3-(3-methoxyphenyl)propanoate (3ib)[3c]:

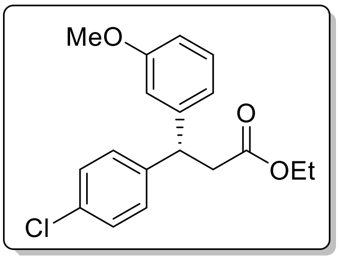

^{1}H NMR ($CDCl_3$, 500 MHz) δ = 1.11 (t, 3H, *J* = 7.1 Hz), 2.94-3.02 (m, 2H), 3.74 (s, 3H), 4.03 (q, 2H, *J* = 7.2 Hz), 4.47 (t, 1H, *J* = 7.9 Hz), 6.72 (d, 2H, *J* = 6.2 Hz), 6.78 (d, 1H, *J* = 7.4 Hz), 7.14-7.24 (m, 5H); ^{13}C NMR ($CDCl_3$, 125 MHz) δ = 14.1, 40.7, 46.4, 55.1, 60.5, 111.7, 113.8, 119.9, 128.7, 129.1, 129.6, 132.4, 141.9, 144.6, 159.8, 171.5. The ee value of the product was determined on Daicel Chiralcel OD-H column with hexane/2-propanol = 99:1, flow = 1.0 mL/min by HPLC analysis. Retention times: 20.6 min [(*S*)-enantiomer], 26.2 min [(*R*)-enantiomer], 99% ee.

Ethyl (*R*)-3-(3-methoxyphenyl)-3-(4-methoxyphenyl)propanoate (3jb)[3c]:

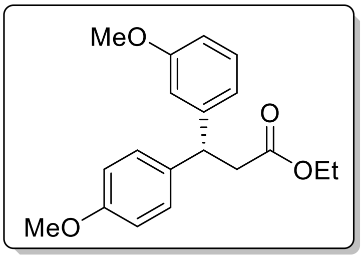

^{1}H NMR ($CDCl_3$, 600 MHz) δ = 1.10 (t, 3H, *J* = 7.2 Hz), 2.98 (d, 2H, *J* = 7.6 Hz), 3.74 (s, 6H), 4.02 (q, 2H, *J* = 7.1 Hz), 4.45 (t, 1H, *J* = 8.3 Hz), 6.70 (dd, 1H, *J* = 8.3, 2.8 Hz), 6.75 (d, 1H, *J* = 2.1 Hz), 6.79-6.80 (m, 3H), 7.13-7.18 (m, 3H); ^{13}C NMR ($CDCl_3$, 125 MHz) δ = 14.1, 41.0, 46.3, 55.1, 55.2, 60.4, 111.4, 113.7, 113.9, 119.9, 128.6, 129.4, 135.5, 145.5, 158.2, 159.6, 171.8. The ee value of the product was determined on Daicel Chiralpak AS-H column with hexane/2-propanol = 99:1, flow = 1.0 mL/min by HPLC analysis. Retention times: 25.5 min [(*R*)-enantiomer], 31.8 min [(*S*)-enantiomer], 98% ee.

Ethyl (*S*)-3-(3-methoxyphenyl)-3-(naphthalen-1-yl)propanoate (3kb)[3c]:

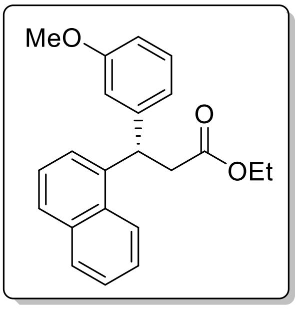

^{1}H NMR ($CDCl_3$, 500 MHz) δ = 1.09 (t, 3H, *J* = 7.1 Hz), 3.10-3.20 (m, 2H), 3.71 (s, 3H), 4.04 (q, 2H, *J* = 7.0 Hz), 5.36 (t, 1H, *J* = 7.9 Hz), 6.70 (dd, 1H, *J* = 7.9, 2.3 Hz), 6.82 (d, 1H, *J* = 2.0 Hz), 6.88 (d, 1H, *J* = 7.9 Hz), 7.17 (dd, 1H, *J* = 7.8 Hz, 2.8 Hz), 7.39-7.47 (m, 4H), 7.73 (d, 1H, *J* = 7.9 Hz), 7.81-7.83 (m, 1H), 8.15 (d, 1H, *J* = 8.5 Hz); ^{13}C NMR ($CDCl_3$, 125 MHz) δ = 14.0, 41.3, 42.6, 55.1, 60.5, 111.5, 114.0, 120.3, 123.7, 124.2, 125.2, 125.5, 126.1, 127.4, 128.8, 129.5, 131.6, 134.1, 138.8, 145.1, 159.7, 171.8. The ee value of the product was determined on Daicel Chiralcel OD-H column with hexane/2-propanol = 99:1, flow = 1.0 mL/min by HPLC analysis. Retention times: 35.2 min [(*S*)-enantiomer], 66.4 min [(*R*)-enantiomer], 99% ee.

Ethyl (*S*)-3-(furan-2-yl)-3-(3-methoxyphenyl)propanoate (3lb)[3c]:

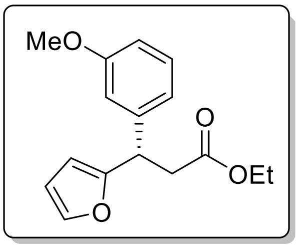

^{1}H NMR ($CDCl_3$, 500 MHz) δ = 1.14 (t, 3H, *J* = 7.4 Hz), 2.86 (dd, 1H, *J* = 15.6, 7.6 Hz), 3.05, (dd, 1H, 15.9, 7.9 Hz), 3.76 (s, 3H), 4.06 (q, 2H, *J* = 7.2, Hz), 4.51 (t, 1H, *J* = 7.9 Hz), 6.04 (d, 1H, *J* = 4.0 Hz), 6.25 (s, 1H), 6.75-6.78 (m, 2H), 6.83 (d, 1H, *J* = 7.9 Hz), 7.20 (t, 1H, *J* = 7.9 Hz), 7.29 (t, 1H, *J* = 1.1 Hz); ^{13}C NMR ($CDCl_3$, 125 MHz) δ = 14.1, 39.8, 41.4, 55.2, 60.5, 105.7, 110.1, 112.3, 113.7, 120.1, 129.5, 141.7, 142.8, 156.1, 159.8, 171.3. The ee value of the product was determined on Daicel Chiralcel OD-H column with hexane/2-propanol = 99:1, flow = 1.0 mL/min by HPLC analysis. Retention times: 15.1 min [(*R*)-enantiomer], 17.9 min [(*S*)-enantiomer], 98% ee.

3-2. Leaching test: The reaction was set up by a procedure shown in **3-1**. After the mixture was stirred for 12 h under Ar atmosphere at 100 ˚C, ethylbenzene (15~20 mg) as an internal standard and THF were added to the mixture. The mixture was picked up with a syringe and transferred to a volumetric flask through a membrane filter in order to remove the residual solids and diluted to 10 mL solution by THF. The filtrate (3 mL) was taken by a volumetric pipette and the solvent was removed *in vacuo*. The residual crude mixture was heated in a mixture of sulfuric acid (0.2 mL) and nitric acid at 200 ˚C until all nitric acid was evaporated. The mixture was cooled to room temperature. The solution was diluted to 10 mL solution by pure water, and the resulting solution was measured by ICP analysis to determine the amount of Rh that leached out. Another filtrate (7 mL) and water were transferred to a separating funnel and the aqueous layer was extracted with diethyl ether. The combined organic layers were dried over sodium sulfate and the conversion of **1a** and the yield of (*S*)-5-phenylhexan-3-one **3aa** were determined by GC analysis with reference to an internal standard (IS = ethylbenzene). The residue was purified by preparative TLC to afford **3aa**. The ee value of the product was determined by chiral HPLC analysis.

3-3. Recovery and reuse of the catalyst: (*E*)-Hex-4-en-3-one **1a** (58.8 mg, 0.6 mmol), phenylboronic acid (the mixture of boroxine and boronic acid, 126.7 mg, 1.2 mmol as B), Rh-Cell **III** (Rh: 0.5 mol%), which was heated at 150 ˚C for 2 h just before use, chiral diene **4b** (0.02 mL of 3.9 mg/mL solution of toluene), toluene (0.73 mL) and water (1.5 mL) were combined in a Carousel™ tube. The mixture was stirred for 12 h under Ar atmosphere at 100 ˚C. After the mixture was stirred for 20 h under Ar atmosphere at 100 ˚C, the catalyst was collected by filtration and washed with THF and water using a KIRIYAMAROHTO® funnel. The recovered catalyst was suspended in THF (2 mL) and 1 M TfOH aq. (0.02 mL), and the mixture was stirred at room temperature for 3 h under air. The catalyst was collected by filtration and washed with THF, water and acetone using a KIRIYAMAROHTO® funnel. The recovered catalyst was reused for the next reaction with new portion of substrates and chiral diene. On the other hand, ethylbenzene (15~20 mg) as an internal standard was added to the filtrate of the first filtration, and an aliquot of the mixture (~0.02 mL) was filtered through a silica gel packed disposable Pasteur pipette and washed with ethyl acetate to inject the GC analysis. The conversion of **1a** and the yield of (*S*)-5-phenylhexan-3-one **3aa** were determined by GC analysis with reference to an internal standard (IS = ethylbenzene).

3-4. Preparation of a stock solution of chiral diene with various enantio excess for

examinations of non-linear effects: (*S*)-α-Phellandrene was prepared by following the literature[5] and (*S*,*S*,*S*)-chiral diene **4b** was prepared by the same procedure shown in reference 3c. Stock solutions of (*R*,*R*,*R*)-**4b** and (*S*,*S*,*S*)-**4b** were prepared (3.9 mg/mL in toluene) and these solutions were combined with an appropriate ratio. The ee values of **4b** in each solution were determined on Daicel Chiralcel OD-H column with hexane/2-propanol = 99:1, flow = 0.5 mL/min by HPLC analysis. Retention times: 13.0 min [(*R*)-enantiomer], 14.2 min [(*S*)-enantiomer].

3-5. Reactions to examine non-linear effects (Figure 2): Ethyl 4-methylcinnamate and phenylboronic acid were used as model substrates. The reaction procedure shown in **3-1** was used with Rh-Cell **III** (Rh: 0.5 mol%) and diene **4b** (0.05 mol%). The non-linear effect analysis in homogeneous metal complex system was previously reported (see reference 3c).

Table S1. Failure to prevent the leaching in the reaction with Rh/Cell **7** after several treatments

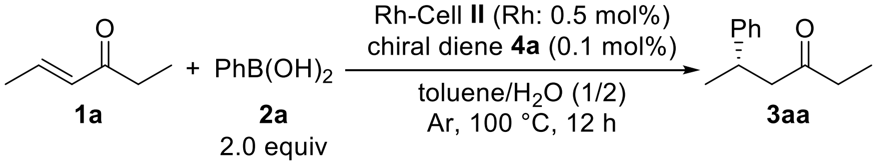

entry	catalyst treatment	yield (%)[a]	leaching (%)[b]
1	-	65	3.0
2	treated with $NaBH_4$ in diglyme	64	3.6
3	NaOH aq. wash	58	7.0
4	heat at 100 ˚C, 1 h, air	96	6.4
5[c]	-	94	9.1
6[c]	heat at 150 ˚C, 3 h, air	85	8.3
7[c]	heat at 150 ˚C, 3 h, in diglyme	43	0.58

[a] Determined by GC analysis. [b] Determined by ICP analysis. [c] The catalyst was prepared diluted conditions (in the reduction step, THF (20 mL) was used, Rh loading: 0.0572 mmol/g).

Table S2. Screening of the amount of the catalyst and the ligand

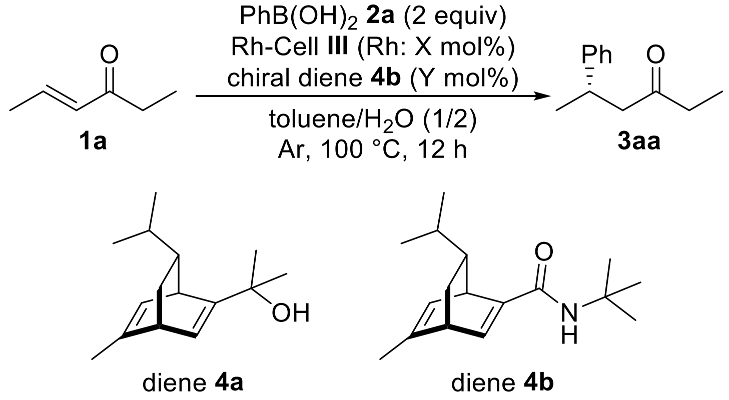

entry	X (mol%)	Y (mol%)	yield (%)[a]	leaching (%)[b]	ee (%)[c]
1	0.5	0.1 (diene **4a**)	86	ND(<0.22)	96
2	0.5	0.1	81	0.85	98
3	0.5	0.05	91	ND(<0.17)	98
4	0.25	0.05	33	ND(<0.37)	-
5	0.5	0.025	35	ND(<0.18)	-

[a] Determined by GC analysis. [b] Determined by ICP analysis. [c] Determined by HPLC analysis.

Table S3. Order of the addition of reagents during catalyst preparation (no pre-stirring)

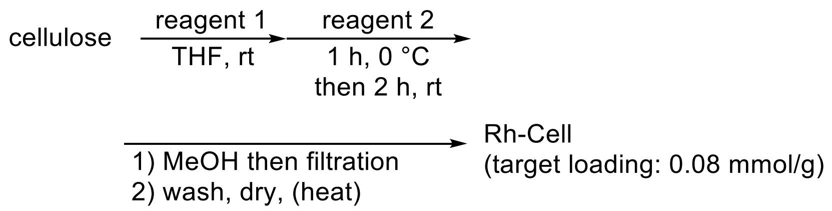

entry	reagent 1	reagent 2	Rh loading (mmol/g)[a]
1	$NaBH_4$ (20 equiv)	$Rh_2(OAc)_4$	0.0453
2	$Rh_2(OAc)_4$	$NaBH_4$ (20 equiv)	0.0376

[a] Determined by ICP analysis.

4. Structure analysis

4-1. Control study to clarify the interaction between Rh NPs and supports

To clarify the interaction between Rh NPs and cellulose, control studies with partially methylated cellulose (MeCell) and D-glucose (Glu) as supports were conducted. As these materials can dissolve in water and/or methanol, the catalysts were prepared without using water and methanol (Table S4). Comparing with Rh-Cell **V** that was prepared by this revised method (entry 1), similar loadings were obtained in both Rh-MeCell (entry 2) and Rh-Glu (entry 3), indicating that even the supports with less amounts of hydroxyl groups or no polymer matrix could stabilize Rh NPs. STEM images of these catalysts also showed no significant difference and small NPs (~3 nm) were well stabilized. Moreover, IR analysis of **V** and Rh-Glu showed no significant change of the IR spectrum after deposition of Rh NPs. Judging from these results, there was no formation of strong bond, such as covalent bonds or hydrogen bonds between Rh NPs and the supports, and Rh NPs might be stabilized by interactions with oxygen atoms of cellulose.

Table S4. Preparation of polysaccharide-supported Rh NP catalysts

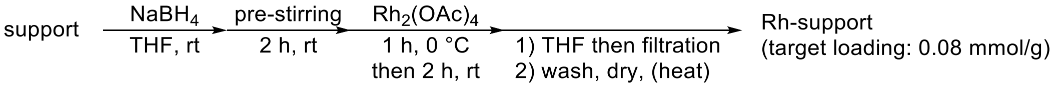

entry	catalyst	reagents	Rh loading (mmol/g)[a]
1	Rh-Cell **V**	cellulose	0.0629
2	Rh-MeCell	partially methylated cellulose	0.0595
3	Rh-Glu	D-Glucose	0.0669

[a] Determined by ICP analysis.

4-2. STEM images

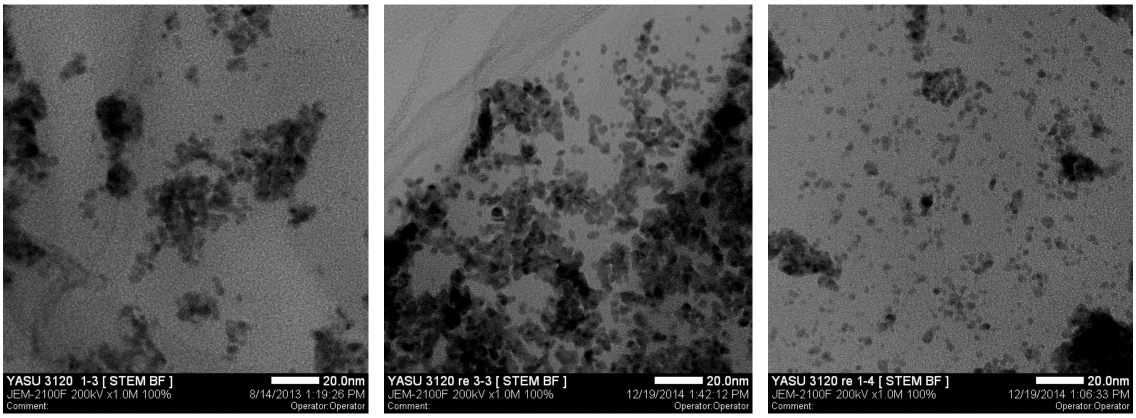

Figure S1. STEM images of Rh-Cell **I**

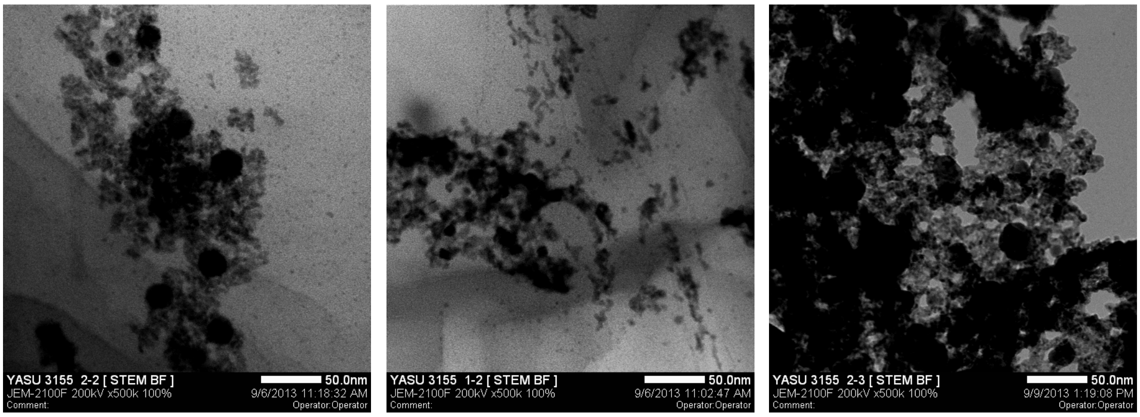

Figure S2. STEM images of Rh/Ag-Cell

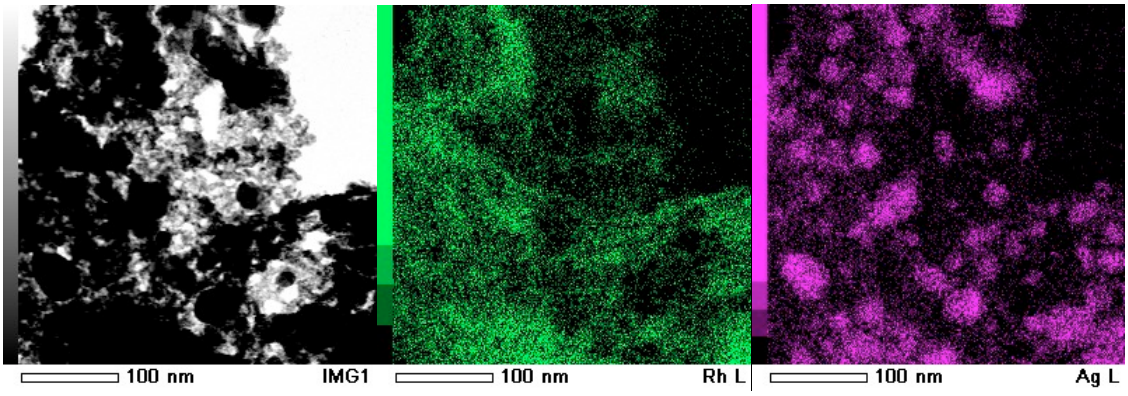

Figure S3. EDS mappings of Rh/Ag-Cell

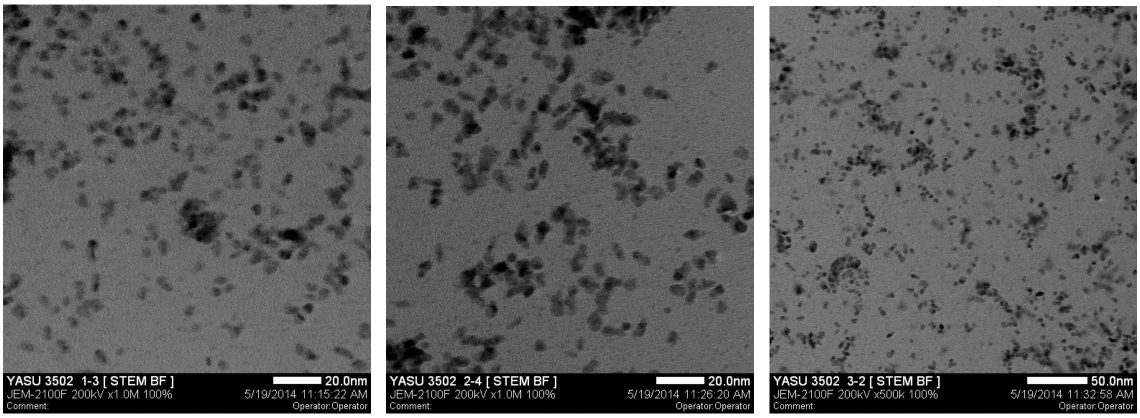

Figure S4. STEM images of Rh-Cell **II**

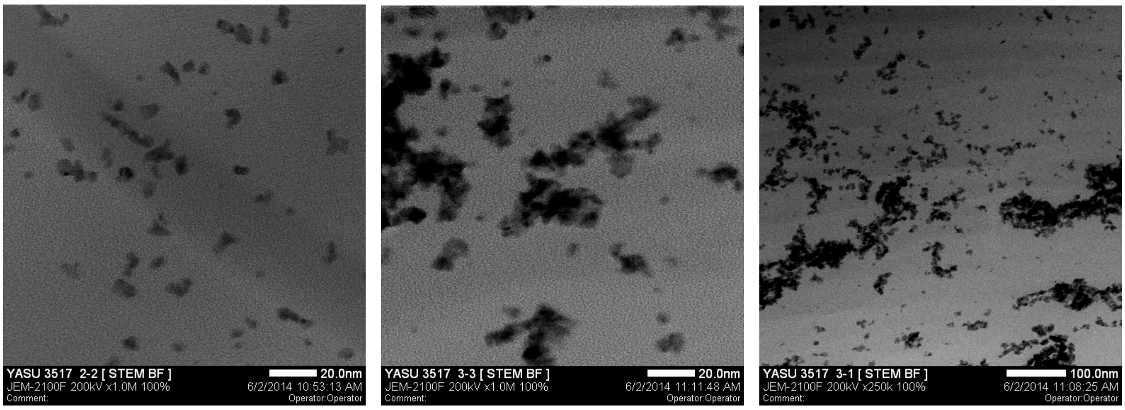

Figure S5. STEM images of Rh-Cell **III**

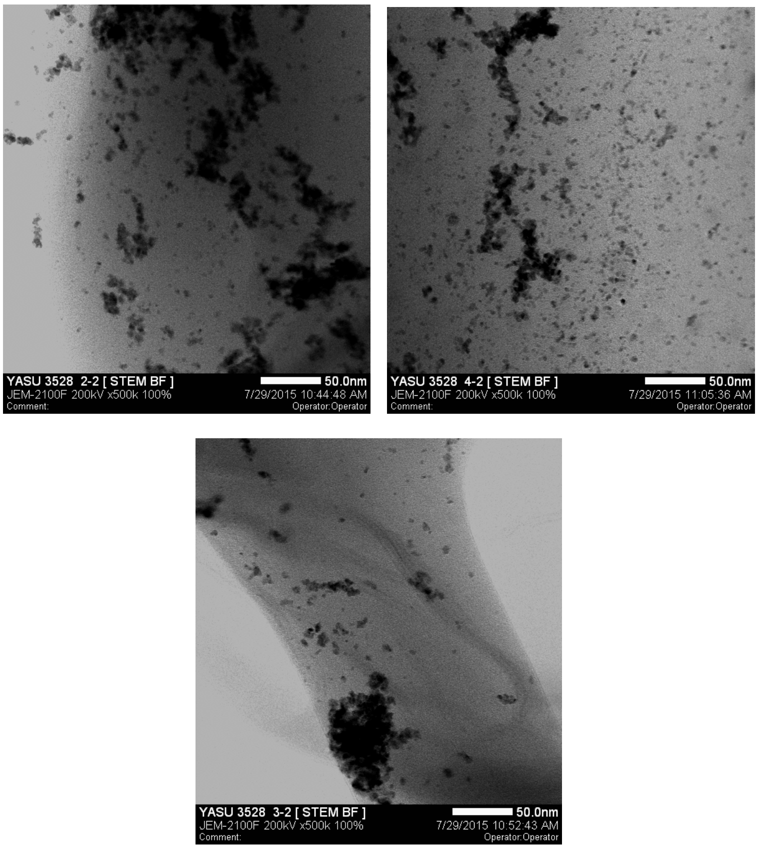

Figure S6. STEM images of Rh-Cell **IV**

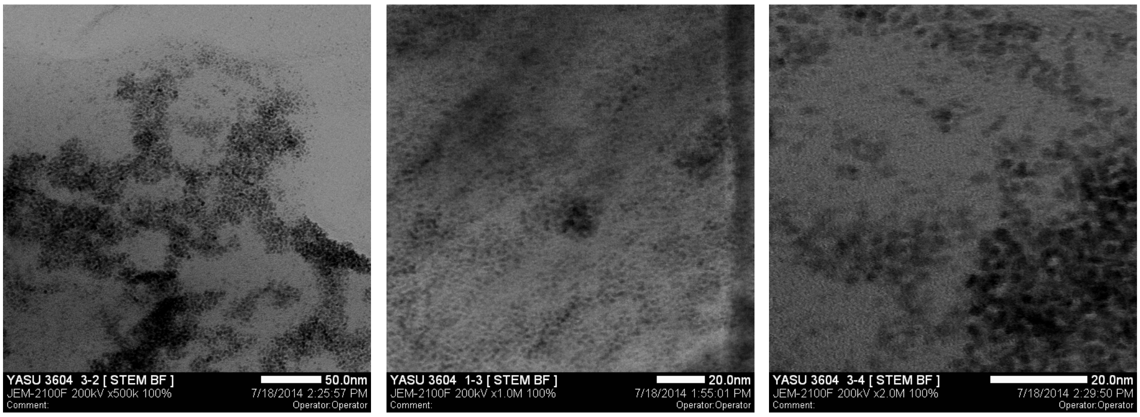

Figure S7. STEM images of Rh-MeCell

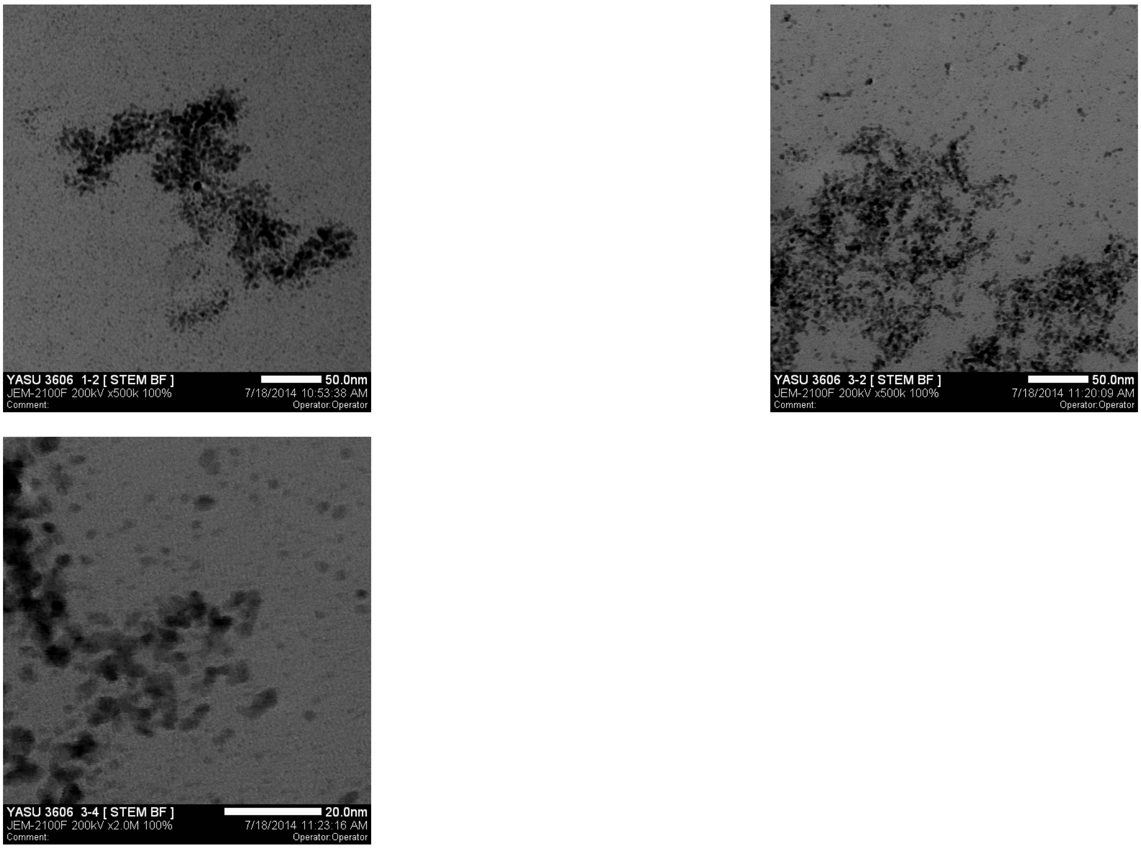

Figure S8. STEM images of Rh-Glu

4-3. Preparation of sugar or polysaccharide-supported catalysts without using methanol and water (Table S4): To a suspension of D-glucose (1.2 g) in THF (10 mL), a solution of $NaBH_4$ (72.6 mg, 1.92 mmol) in diglyme (1.5 mL) was added at room temperature, and the mixture was stirred for 2 h at the same temperature under Ar atmosphere. To this suspension was slowly added a solution of rhodium(II) acetate

dimer (21.2 mg, 0.048 mmol) in THF (1.5 mL). The mixture was stirred for 1 h at 0 ˚C and the stirring was continued for 2 h at room temperature. The catalyst was filtered, washed with THF and dried *in vacuo* to afford Rh-Glu. Rh-Glu (10-20 mg) was heated in a mixture of sulfuric acid and nitric acid at 200 ˚C, and the mixture was cooled to room temperature. The amount of Rh in the resulting solution was measured by ICP analysis to determine the loading of Rh.

4-4. Preparation of the sample for SR-MAS: Rh/Cell **I** (78.2 mg, the loading of Rh: 0.0639 mmol/g) and chiral diene **4a** (0.1 mL of 2.2 mg/mL in toluene-d_8) were combined in toluene-d_8 (0.2 mL), and the mixture was stirred at room temperature under Ar atmosphere for 30 minutes. Excess toluene was removed *in vacuo* and the swelled solids were transferred to a sample tube. The pulse sequence including diffusion filter and isotropic mixing was shown in Scheme S1.

Scheme S1. Pulse sequence of diffusion filter and isotropic mixing

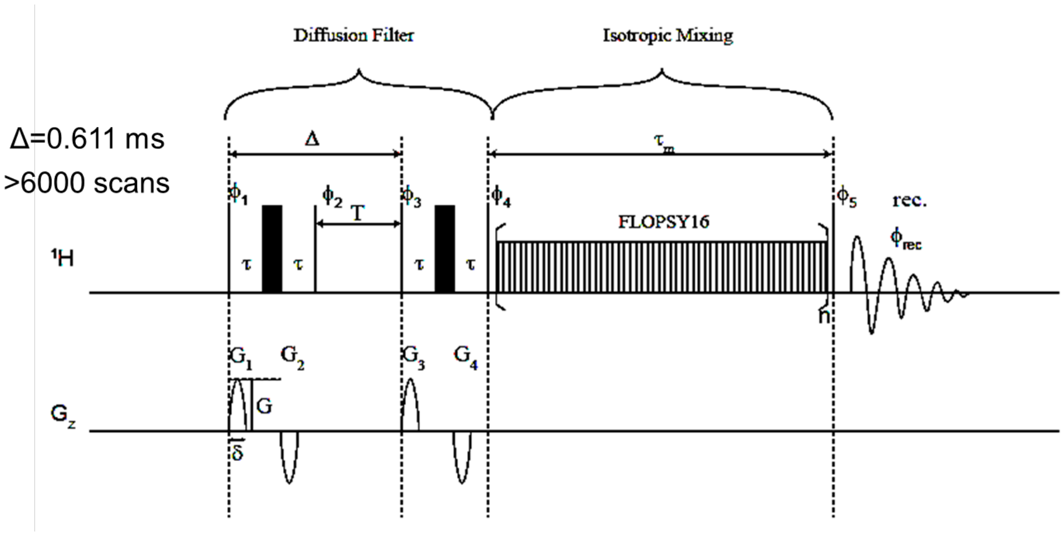

The initial induced proton spins of mobile molecules (molecules in bulk) are suppressed by applying stimulated-echo diffusion filter. The proton spin transfer between polymer-ligand is achieved by the isotropic mixing with FLOPSY16 loop. Narrow and Wide bars depict 90° and 180° pulses, respectively. All pulses are x phase unless otherwise indicated.

The delays are Δ = 0.611 ms (should be multiple of the inverse of Sample Spinning Speed); T = 0.311 ms ; τ_m = 7.85 ms(n=1) or 15.7 ms(n=2); τ = 100 μs; δ = 50 μs, the phase cycling is φ_1 = x,-x; φ_2 = -x, x; φ_3 = x, -x; φ_4 = x, x, x, x, -x, -x, -x, -x; φ_5 = x, x, -x, -x; φ_{rec} = x, -x, -x, x, -x, x, x, -x and the amplitudes $G_1 = -G_2 = G_3 = -G_4 = G$.

The FLOPSY16 loop is composed from 16 blocks that block contains 9 pulses denoted as follows.

The pulses are written with flip angles and phases in degree (e.g. 46(0) denotes the pulse width corresponds to the 46 degree pulse and whose phase is zero degree), and where the overbar indicates a phase shift of all constituent pulses by 180°.

FLOPSY16 = $R\bar{R}\bar{R}R$ $RR\bar{R}\bar{R}$ $\bar{R}RR\bar{R}$ $\bar{R}\bar{R}RR$

R = 46(0) 96(45) 164(67.5) 159(315) 130(22.5) 159(315) 164(67.5) 96(45) 46(0);

4-5. SR-MAS study (control experiments)

In a sample tube, 1:5 ratio of the diene and Rh was combined. When the spectrum was taken by single pulse, many signals derived from the diene, cellulose and the solvent were detected after 5000 scans (Figure S9). When the SR-MAS study was conducted with diffusion filter, the signals derived from the diene at 3-6 ppm were completely suppressed after more than 6000 scans while the broad signal derived from cellulose around 5 ppm remained (Figure 2a, the main text). The signals derived from

the solvent could not be completely suppressed even with diffusion filter under this condition. The broad signal of cellulose was completely suppressed and alternatively, the signals of the diene appeared again with the combination of diffusion filter and isotropic mixing when the mixing time was 7.85 ms (Figure 2b, the main text). In the case of the shorter mixing time, a similar chart was obtained (Figure S10) while the signals of the diene did not appear strongly in the case of the longer mixing time (Figure S11). In all cases, the signals of the solvent still remained and the signal of cellulose was not detected. If the signals of the diene appear by the magnetization transfer from the remaining solvent, the signals of the diene would appear even in the case of the longer mixing time. However, the signals of the diene was not enhanced when the mixing time was 15.7 ms. Judging from these results, these diene signals might appear by the magnetization transfer from cellulose. As control, a mixture of cellulose and diene **4a** was analyzed by the same method. The signals of the diene could not be suppressed even with diffusion filter while the signal of cellulose completely diminished due to very short relaxation time under this condition (Figure S12). The concentration of the diene in the solution phase might be too high to eliminate their signals completely. As the signals of the diene could be suppressed in the case of the experiments with Rh/Cell, it was implied that the mobility of the most chiral diene might become low in the presence of Rh nanoparticles. From these results, it might be said that molecules adsorbed on Rh nanoparticles were selectively observed by this method.

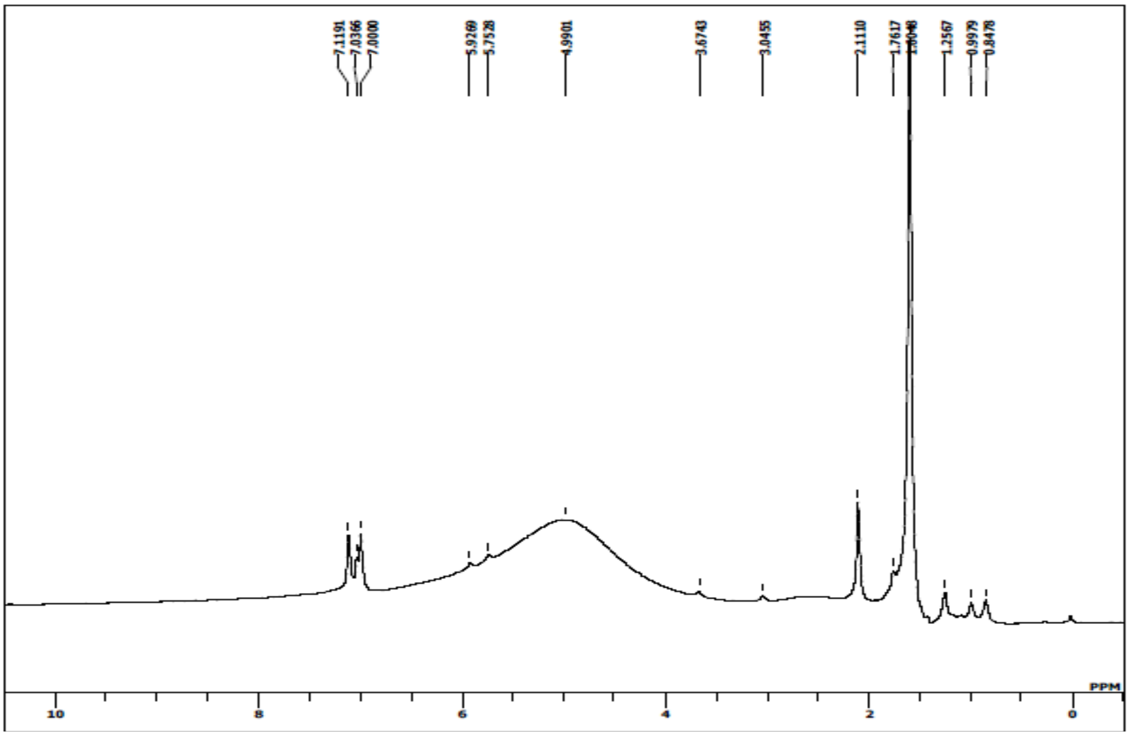

Figure S9. SR-MAS study (single pulse)

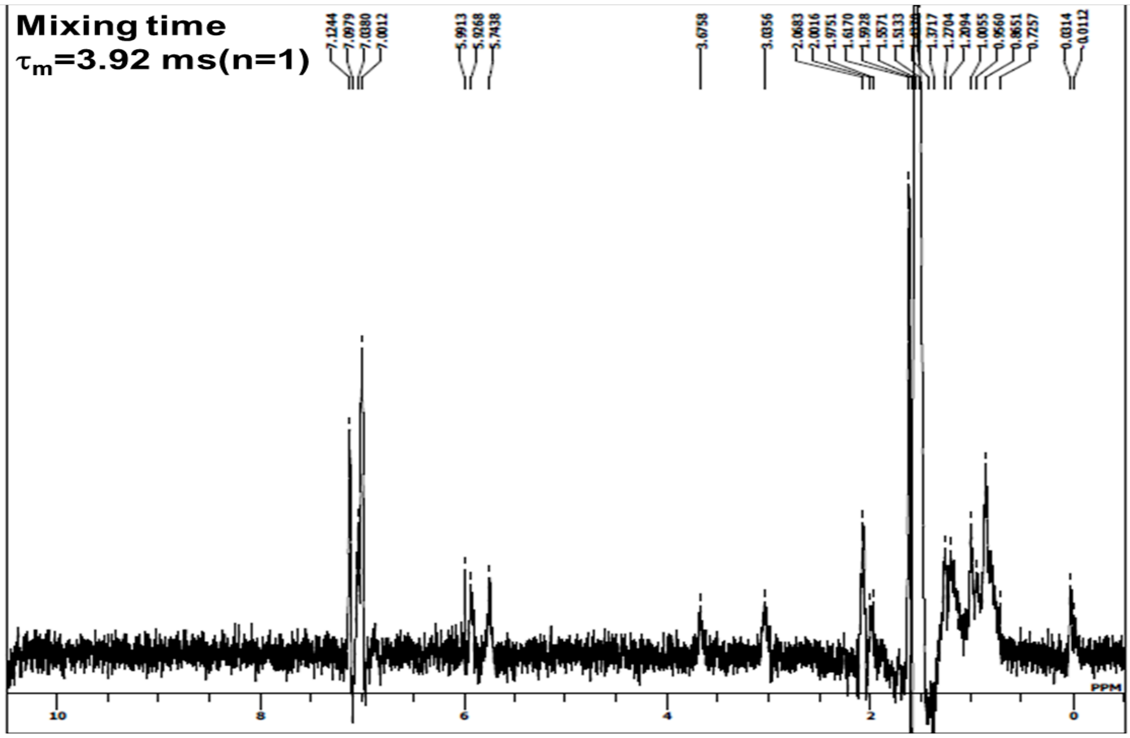

Figure S10. SR-MAS study with isotropic mixing (mixing time: 3.92 ms)

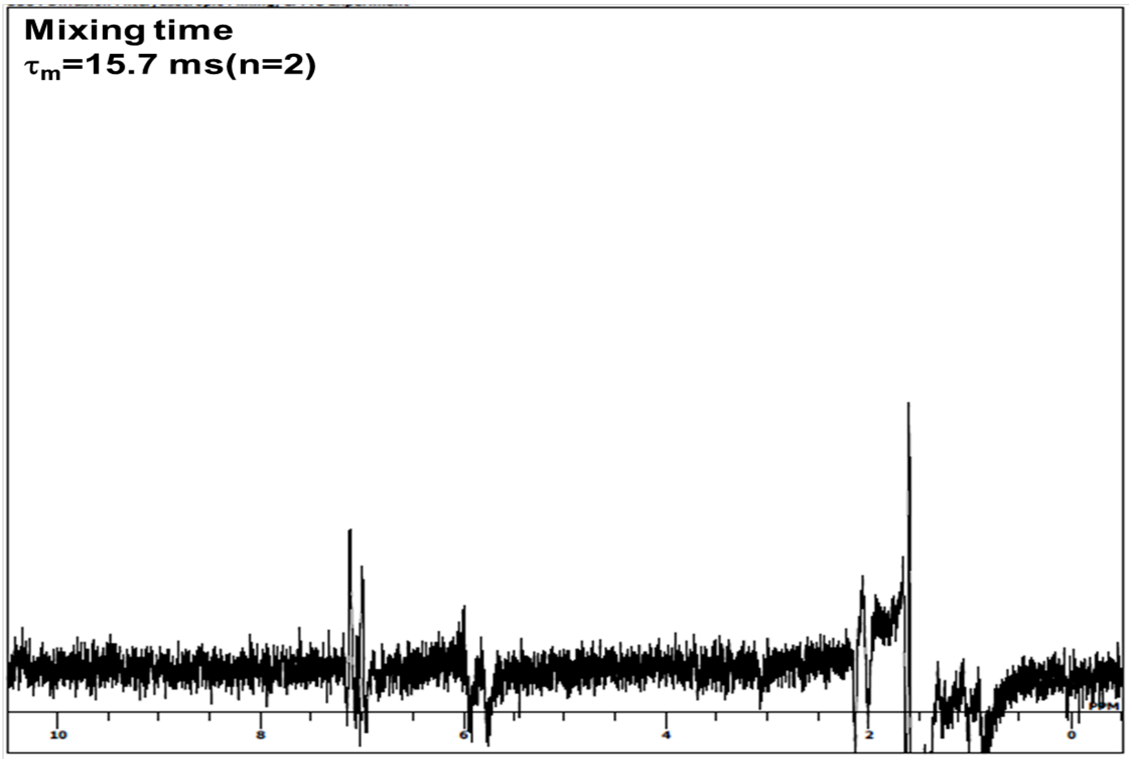

Figure S11. SR-MAS study with isotropic mixing (mixing time: 15.7 ms)

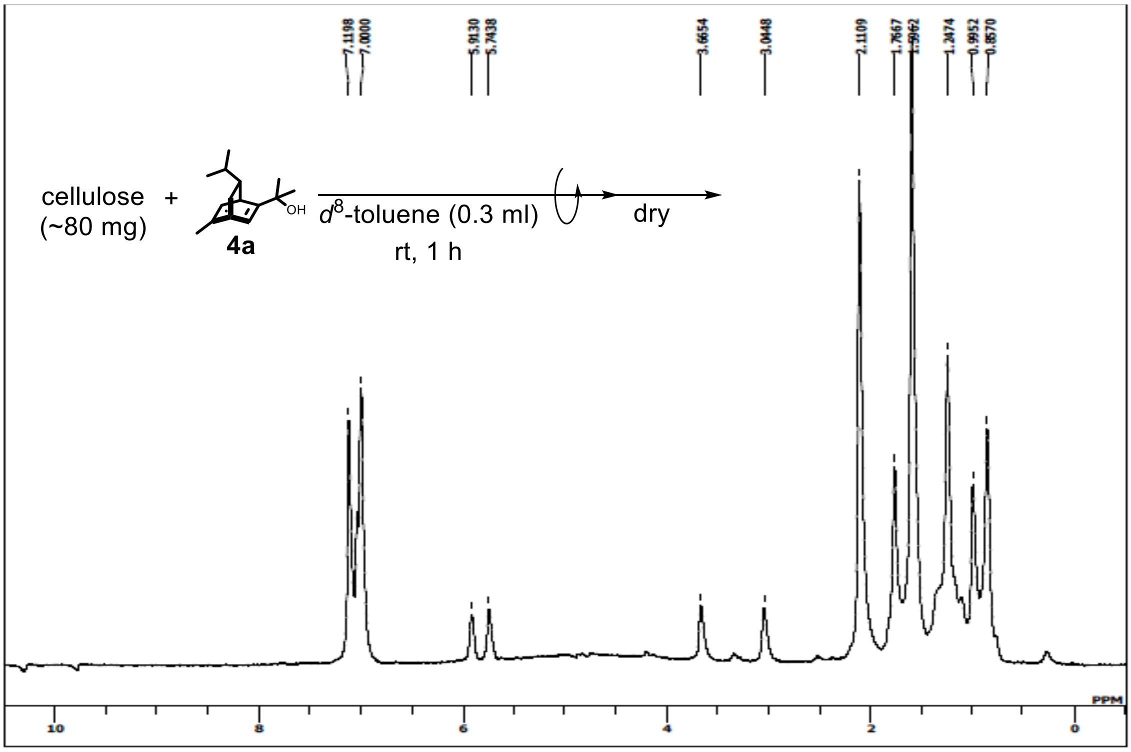

Figure S12. Control experiment without Rh (SR-MAS study with diffusion filter)

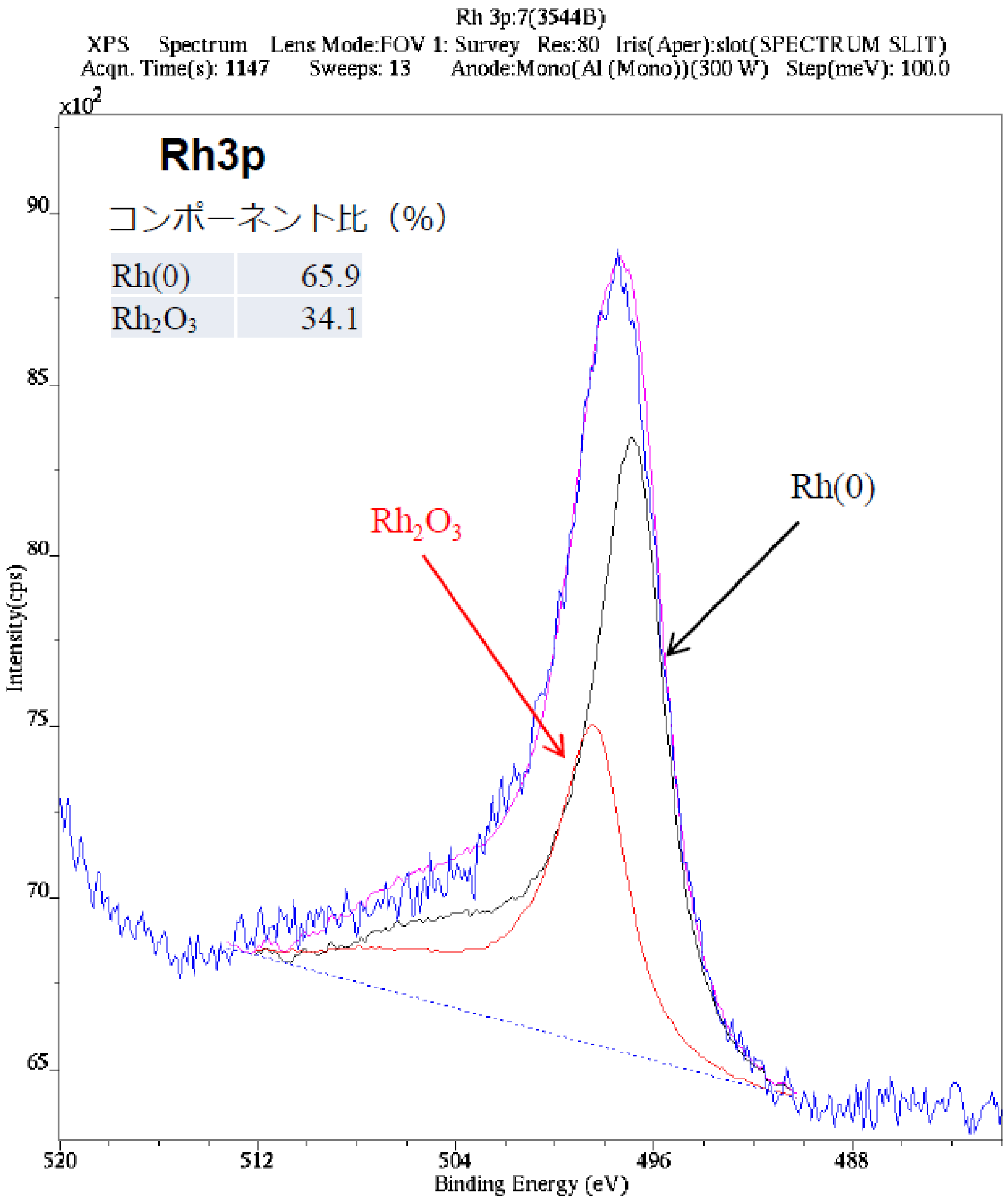

Figure S13. XPS analysis of Rh-Cell **III**

5. References

[1] H. C. Brown, E. J. Mead, B. C. S. Rao, *J. Am. Chem. Soc.* **1955**, *77*, 6209.

[2] (a) B. List, A. Doehring, M. T. H. Fonseca, K. Wobser, H. V. Thiene, R. R. Torres, P. L. Galilea, *Adv, Synth. Catal.* **2005**, *347*, 1558; (b) B. List, A. Doehring, M. T. Hechavarria Fonseca, A. Job, R. Rios Torres, *Tetrahedron* **2006**, *62*, 476.

[3] For **4a**: (a) K. Okamoto, T. Hayashi, V. H. Rawal, *Chem. Commun.* **2009**, 4815; (b) K. Okamoto, T. Hayashi, V. H. Rawal, *Org. Lett*. **2008**, *10*, 4387; For **4b**: (c) T. Yasukawa, A. Suzuki, H. Miyamura, K. Nishino, S. Kobayashi, *J. Am. Chem. Soc.* **2015**, *137*, 6616.

[4] T. Yasukawa, H. Miyamura, S. Kobayashi, *J. Am. Chem. Soc.* **2012**, *134*, 16963.

[5] A. Sen, W. Grosch, *Flavour and Fragrance J.* **1990**, *5*, 233.
